# Phosphate rebinding induces force reversal via slow backward cycling of cross-bridges

**DOI:** 10.3389/fphys.2024.1476876

**Published:** 2025-01-07

**Authors:** Robert Stehle

**Affiliations:** Institute of Vegetative Physiology, University of Cologne, Köln, Germany

**Keywords:** cross-bridge cycle, cross-bridge model, phosphate release, phosphate binding, tension redevelopment, cardiac myofibrils, muscle force generation, rate limiting steps

## Abstract

**Objective:**

Previous studies on muscle fibers, myofibrils, and myosin revealed that the release of inorganic phosphate (P_i_) and the force-generating step(s) are reversible, with cross-bridges also cycling backward through these steps by reversing force-generating steps and rebinding P_i_. The aim was to explore the significance of force redevelopment kinetics (rate constant *k*_TR_) in cardiac myofibrils for the coupling between the P_i_ binding induced force reversal and the rate-limiting transition *f* ^–^ for backward cycling of cross-bridges from force-generating to non-force-generating states.

**Methods:**

*k*_TR_ and force generation of cardiac myofibrils from guinea pigs were investigated at 0.015–20 mM P_i_. The observed force-[P_i_], force-log [P_i_], *k*_TR_-[P_i_], and *k*_TR_-force relations were assessed with various single-pathway models of the cross-bridge cycle that differed in sequence and kinetics of reversible P_i_ release, reversible force-generating step and reversible rate-limiting transition. Based on the interpretation that *k*_TR_ reflects the sum of rate-limiting transitions in the cross-bridge cycle, an indicator, the coupling strength, was defined to quantify the contribution of P_i_ binding induced force reversal to the rate-limiting transition *f* ^–^ from the [P_i_]-modulated *k*_TR_-force relation.

**Results:**

Increasing [P_i_] decreased force by a bi-linear force-log [P_i_] relation, increased *k*_TR_ in a slightly downward curved dependence with [P_i_], and altered *k*_TR_ almost reciprocally to force reflected by the *k*_TR_-force relation. Force-[P_i_] and force-log [P_i_] relations provided less selectivity for the exclusion of models than the *k*_TR_-[P_i_] and *k*_TR_-force relations. The *k*_TR_-force relation observed in experiments with cardiac myofibrils yielded the coupling strength +0.84 ± 0.08 close to 1, the maximum coupling strength expected for the reciprocal *k*_TR_–force relationship. Single pathway models consisting of fast reversible force generation before or after rapid reversible P_i_ release failed to describe the observed *k*_TR_–force relation. Single pathway models consistent with the observed *k*_TR_-force relation had either slow P_i_ binding or slow force reversal, i.e., in the consistent single pathway models, *f* ^–^ was assigned to the rate of either P_i_ binding or force reversal.

**Conclusion:**

Backward flux of cross-bridges from force-generating to non-force-generating states is limited by the rates of P_i_ binding or force reversal ruling out other rate-limiting steps uncoupled from P_i_ binding induced force reversal.

## 1 Introduction

Muscle generates force through the cross-bridge ATPase cycle, during which the cross-bridges pass through various chemical and structural states, which are grouped into non-force-generating and force-generating states. The forward transition from non-force-generating to force-generating states is associated with the release of inorganic phosphate (P_i_) and the force-generating step, also called the power stroke when referring to the individual myosin motor. Studies on muscle fibers and myosin working under load indicate that P_i_ release is reversible, allowing cross-bridges to rebind P_i_ and reverse force generation, i.e., the force produced by cross-bridges, by cycling backward from force-generating to non-force-generating states (Mannherz, 1970; Ulbrich and Ruegg, 1971; Hibberd et al., 1985a; Hibberd et al., 1985b; Webb et al., 1986). However, models of the cross-bridge ATPase cycle differ with respect to the sequence of P_i_ release and the power stroke. The original model, which proposed that the force-generating step occurs concurrently with P_i_ release (Eisenberg et al., 1980), has undergone continuous refinement. Many studies support models where the power stroke precedes P_i_ release (Millar and Homsher, 1990; Kawai and Halvorson, 1991; Dantzig et al., 1992; Ranatunga, 1999; Muretta et al., 2015; Woody et al., 2019; Matusovsky et al., 2021; Scott et al., 2021), whereas others support the opposite sequence (Davis and Rodgers, 1995; Smith, 2014; Llinas et al., 2015; Rahman et al., 2018; Offer and Ranatunga, 2020; Hwang et al., 2021; Moretto et al., 2022). Further complexity arises because of the need for at least one additional power stroke (Capitanio et al., 2006; Hwang et al., 2021; Matusovsky et al., 2021) and recent evidence from kinetics of single molecule fluorescence and molecular modelling that P_i_ release from muscle myosin (myosin II) occurs in multiple step (Moretto et al., 2022). Earlier evidence for a stepwise mechanism of P_i_ release has been given by studies of crystal structures of myosin VI (Llinas et al., 2015; Robert-Paganin et al., 2020). Moreover, the correlation between mechanical and energetic quantities led to the concept that the myosin power stroke is weakly coupled to its ATPase cycle (Yanagida et al., 1985; Ishijima et al., 1998) or more concretely to models in which cross-bridges can cycle through additional pathways, enabling some uncoupling (Linari et al., 2010; Debold et al., 2013; Scott et al., 2021), or loosening of the coupling (Caremani et al., 2013; Governali et al., 2020) between force generation and P_i_ release. Additional pathways include the detachment of myosin from actin before releasing P_i_ (Linari et al., 2010), P_i_ release from one pre-power stroke state and three post-power stroke states (Caremani et al., 2013; Governali et al., 2020), and detachment of myosin upon rebinding of P_i_ to the pre-power stroke (Debold et al., 2013) or to the post-power stroke state (Scott et al., 2021). The advantages and disadvantages of the different models are discussed in details (Debold, 2021; Mansson et al., 2023; Rassier and Mansson, 2025); however, arriving at a consensus remains challenging to date.

Understanding the mechanisms of P_i_ release and force generation is linked to their relation to the transitions limiting the rates of cross-bridge cycling. Although these rate-limiting transitions have been explored in many studies discussed by Gordon et al. (2000), Takagi et al. (2004), Geeves and Holmes (2005), Mansson et al. (2015), Stehle and Tesi (2017), Rahman et al. (2018), their nature remains controversial. Elucidating the relationship between these rate-limiting transitions and reversible P_i_ release and force generation is important for developing targeted strategies to modulate the rate of muscle contraction reviewed by Gordon et al. (2000), Takagi et al., (2004), Hinken and Solaro (2007), Stehle and Iorga (2010), Mansson et al. (2015), Geeves (2016), Houdusse and Sweeney (2016), Stehle and Tesi (2017). This study aims to explore the coupling between the process of P_i_ release-associated force generation and rate-limiting transitions. This coupling is still poorly understood, even for the main pathway. Because of the open questions regarding the sequence of P_i_ release and force generation and the increasing difficulty to identify specific rate-limiting transitions in multi-step and multi-pathway model, the strategy for defining the constraints for the rate-limiting step in this study was to analyze simple, single-pathway models with various sequence and kinetics of the P_i_ release and the force-generating step.

A measurement of the rates limiting the transition between non-force-generating and force-generating states is the kinetics of mechanically-induced force redevelopment induced by rapidly switching from a transient period of active unloaded shortening to active isometric contraction (Brenner, 1988). The rate constant *k*_TR_ of this force redevelopment represents the sum of apparent rate constants in the cross-bridge ATPase cycle limiting the transitions of cross-bridges between non-force-generating and force-generating states (Brenner, 1988), reviewed in Gordon et al. (2000). The addition of P_i_ increases *k*_TR_ and decreases force in skeletal and cardiac muscle (Millar and Homsher, 1990; Regnier et al., 1995; Araujo and Walker, 1996; Wahr et al., 1997; Regnier and Homsher, 1998; Tesi et al., 2000; Stehle et al., 2002a; Tesi et al., 2002; Hinken and McDonald, 2004; Edes et al., 2007; Papp et al., 2014; Stehle, 2017; Stehle and Tesi, 2017; Governali et al., 2020). The opposing effects of [P_i_] on *k*_TR_ and force provide evidence for P_i_ shifting of cross-bridges backward from force-generating to non-force-generating states (Hibberd et al., 1985a; Hibberd et al., 1985b; Webb et al., 1986; Kawai and Halvorson, 1991; Dantzig et al., 1992; Ranatunga, 1999; Mansfield et al., 2012; Woodward and Debold, 2018). Without P_i_, redistribution is solely determined by the rate-limiting forward transitions *f* and *g* in the ATPase cycle, and *k*_TR_ = *f* + *g,* with *f* denoting the apparent rate constant of the transition to force-generating states and *g* the apparent rate constant of the transition to non-force-generating states. Increasing [P_i_] promotes the rebinding of P_i_ and facilitates the backward transition to non-force states, characterized by the apparent rate constant *f* ^–^ which contributes to *k*_TR_ by *k*_TR_ = *f* + *g* + *f* ^–^, where *f* ^–^ is a function of [P_i_.] (Palmer and Kentish, 1998; Stehle et al., 2002a; Stehle and Tesi, 2017). Recently, the reversibility of force generation was demonstrated at the molecular level by identifying elementary reverse strokes of force measurements on single cardiac myosin heads and filaments (Woody et al., 2019; Hwang et al., 2021). The step size of the two reverse strokes quantified in the experiments with single cardiac myosin were −6 nm and −3 nm and of similar magnitude as the two forward strokes of +6 nm and +3 nm (Hwang et al., 2021). Importantly, reverse strokes were confirmed by their experiments with single cardiac myosin filaments also to occur at high physiological [ATP].

The present study explored the effects of [P_i_] on *k*_TR_ and force in cardiac myofibrils of guinea pigs on the kinetic coupling of P_i_ binding induced force reversal and *f* ^–^. The interrelation between *k*_TR_ and force at various [P_i_], i.e., the P_i_-modulated *k*_TR_-force relation is demonstrated to provide a basis for probing the strength of this coupling. The coupling strength (*CS*) derived from this interrelation reaches its theoretical maximum when P_i_ binding and the reversal of the force-generating step are combined with *f* ^*–*^ into a slow single step, according to the limiting case of a two-state cross-bridge model in which P_i_ alters *k*_TR_ and force in a simple reciprocal manner, such that, *k*_TR_ changes in proportion to 1/force, i.e., delta *k*_TR_ = 1/delta *F*. Based on this limiting case, an empirical equation was developed to define *CS* on a scale of +1 for maximum coupling and 0 for the case when *k*_TR_ becomes independent of [P_i_]. The P_i_-modulated *k*_TR_-force relation of the cardiac myofibrils from guinea pigs yielded a high *CS* of +0.84 ± 0.08. Testing different models revealed that either P_i_ binding or force reversal, or both, must be connected to *f* ^*–*^ to yield the high *CS* observed in the experiments.

## 2 Materials and methods

### 2.1 Myofibrillar preparation and solutions

Dunkin-Hartley guinea pigs weighing 450–750 g were anesthetized with 5 vol% isoflurane and euthanized by decapitation. The use of animals and procedures in this study complied with the law for animal protection (TierSchG) transferred from the EU guidelines and was reviewed and approved by the Official Animal Care and Use Committee (LANUV NRW, Az 84-02.05.20.13.080 and 84-02.05.50.15.029). After exsanguination of the animal body, the heart was excised, and skinned strips from the trabeculae were prepared as described previously (Linke et al., 1993). First, the blood was removed from the heart by a brief (2–3 min) retrograde perfusion through the aorta at 37°C using a perfusion solution containing 132 mM NaCl, 5 mM KCl, 1 mM MgCl_2_, 10 mM TRIS, 5 mM EGTA, 1 mM sodium azide, 7 mM glucose, and 2 mM DTT, adjusted to pH 7.1. The heart was then transferred into an ice-cold perfusion solution without glucose, and the left ventricular cavity was opened by cutting in the axial direction. Thin strips with diameters of 0.3–0.4 mm were dissected from the endocardial *trabeculae carneae* under observation through a Olympus SZ51 stereomicroscope (Olympus, Hamburg, Germany) at about 20-fold magnification using a Vannas spring Scissors and a Dumont#5SF forceps (Fine Science Tools, Heidelberg, Germany) and pinned with microneedles on the Sylgard surface (Sylgard 184 Elastomer Kit, Dow Corporate, Mat. No. 4019862) in a chamber containing ice-cold skinning solution comprising 1% v/v Triton-X-100, 5 mM K-phosphate, 5 mM Na-azide, 3 mM Mg-acetate, 5 mM K_2_EGTA, 3 mM Na_2_ATP (including 3 mM MgCl_2_ and 6 mM KOH), 47 mM Na_2_CrP, 2 mM DTT, 0.5 mM 4-(2-aminoethyl) benzenesulfonylfluoride HCl, 10 μM leupeptin, 10 μM antipaine, 5 mg/mL aprotinine (adjusted to pH 7 at 0°C). EGTA (324,626) was from Merck Millipore. Triton X-100 (T8787), Na_2_ATP (A2383), Na_2_CrP (2,380) and protease inhibitors were from Merck Sigma Aldrich in high purity grade. The pinned strips were incubated in the skinning solution at 0°C for 4 h, and the skinning solution was replaced by storage solution (same composition as the skinning solution but without triton), in which the skinned strips were stored at 4°C for up to 3 days. Myofibrils were prepared on the day of the mechanical experiment by homogenizing the skinned strips at 0°C for 4–6 s at maximum speed using a blender (T10 Ultra-Turrax, IKA, Stauffen, Germany). The homogenate was then filtered through polypropylene meshes with 22 µm pore openings.

The standard activating buffer (pCa 4.5) used for mechanical experiments contained 10 mM imidazole, 3 mM CaCl_2_K_4_EGTA, 1 mM Na_2_MgATP, 3 mM MgCl_2_, 47.7 mM Na_2_CrP, 2 mM DTT, and different [P_i_] with a pH of 7.0 at 10°C, and µ = 0.178 M. The standard relaxation buffer (pCa 7) contained 3 mM K_4_Cl_2_EGTA, instead of 3 mM CaCl_2_K_4_EGTA. Submaximal activating buffers, pCa (5.88–5.03), were prepared by mixing the standard activating and relaxing buffers in different ratios. Free calcium concentration [Ca^2+^] and pCa = -log [Ca^2+^]/M were calculated using a computer program (Fabiato and Fabiato, 1979). The [P_i_] in the buffers was measured using a phosphate assay kit (E−6646; Molecular Probes, Eugene, OR). P_i_ contamination in the standard activating buffer was 170 ± 20 µM (mean ± SD). Activating and relaxing buffers of lower [P_i_] (15 ± 5 µM P_i_) were produced by adding 1 mM methylguanosine and 0.5 units/mL purine nucleotide phosphorylase (PNP). Activating and relaxing buffers with higher [P_i_] were produced by adding phosphate buffer (30% NaH_2_PO_4_^−^ 70% Na_2_HPO_4_^2-^, pH 6.85). To maintain constant ionic strength, [Na_2_CrP] was reduced by 0.67 mM per 1 mM increase of [P_i_]. All activating and relaxing buffers of different [P_i_] were adjusted to a final pH 7.0 at 10°C.

### 2.2 Apparatus and technique to measure myofibril force redevelopment

The mechanical setup consisted of an Olympus IX-70 microscope with a self-built rigid stage on top to which all manipulators for positioning of the chamber, the solution flow, the micro-needles and the atomic force cantilever were mounted. The micro-flow for the rapid solution change and the optics for force detection by the principle of atomic force microscopy has been described previously (Stehle et al., 2002a; Stehle et al., 2002b). A droplet of myofibrils suspended in the storage solution was added to the thermostatically controlled (10°C) chamber filled with relaxing solution. After sedimentation, a thin myofibril bundle was picked up from the bottom of the chamber at one of its ends using a tungsten micro-needle (# 5,775, A-M Systems, Inc., Carlsborg, WA) connected via a piezo actuator (P602.1SL, Physik Instr.) to a micromanipulator. The bundle was then moved with the micromanipulator to position its other, free end close to the tip of the atomic force cantilever (Nanoprobe^*©*^ FESP type, compliance: 0.2–0.4 μm/μN), which was coated with a mixture (2:3 v/v) of 4% nitrocellulose in amyl-acetate and silicon adhesive (3140 RTV Coating, Dow Corning, Midland, United States). To fix the free end of the bundle at the surface of the coating, the bundle was pressed against the coating using a microneedle installed on a separate manipulator.

Dimensions and sarcomere length (SL) of myofibrils were determined under phase contrast microscopy using the 60x/0.70 Ph2 LCPlanFl objective and the 1.5 magnification lens built in the IX-70 microscope imaged to an ORCA-ER camera (Hamamatsu Photonics, Japan). The bundles used in the experiments had diameters ranging from 1.0 to 3.2 µm and slack lengths of 31–66 µm. The mean slack sarcomere length was 2.02 ± 0.11 µm (mean ± SD). Prior to activation, the bundles were stretched to a 2.4 µm SL. Signal conditioning for movement of actuators and acquisition of force and length signals was performed using a PCI6110-E device under self-written programs in LabView 4.0 (National Instruments, Austin, TX). During force recording, the myofibrils were exposed to one of two laminar streams of solutions produced by a double-channel theta-style capillary (TGC150-15, Clark Electromed. Instr., UK) and driven by gravitational pressure (30–35 cm H_2_O). Rapid Ca^2+^ activation and relaxation were induced by rapid solution changes (Colomo et al., 1998). The position of the flow was altered by the rapid lateral movement of the capillary, controlled by a piezo actuator (P289.40, Physik Instrumente, Karlsruhe, Germany), which effectively changed the solution at the bundle within 5–15 ms. Force redevelopment (*k*_TR_-measurement) was induced during Ca^2+^ activation. Rapid length changes were applied to the bundle via the microneedle using a piezo actuator (P602.1SL, Physik Instrumente). To determine the rate constant *k*_TR_ for force redevelopment, a single exponential function was fitted to the force transients using the LabView program.

### 2.3 Coupling strength (CS) and model simulation

An indicator, the coupling strength (*CS*) was defined to quantify the coupling between P_i_ binding induced force reduction and the rate-limiting backward transition *f* ^*–*^ in the cross-bridge ATPase cycle. *CS* was scaled to 0 for no coupling, to +1 for maximum coupling, and to approach the limit value of −1 for maximum inverse coupling.

**FIGURE 1:**
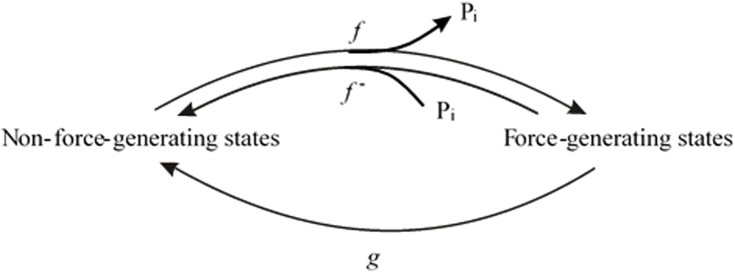
Two-state model in which P_i_ release step, force-generating step and the rate-limiting step for entering force-generating states are merged to single, fully reversible equilibrium.

The maximum positive coupling exists when P_i_ rebinding, the reverse of the force-generating step, and the rate-limiting step for the backward transition of cross-bridges from force-generating states to non-force-generating states all represent the same step, with no other steps contributing to the backward cycling of cross-bridges. This scenario corresponds to the two-state model (Figure 1), involving the forward rates *f* and *g* and the reverse rate *f* ^–^, where *f* represents the P_i_ release-coupled force generation and *f* ^*–*^ the P_i_ binding-coupled reverse of force generation. In this model, the force (*F*) is proportional to the fraction of attached motors *f*/(*f* + *g* + *f* ^–^), and the rate constant of tension redevelopment *k*_TR_ is equal to the sum of the rate constants: *k*_TR_ = *f* + *g* + *f* ^–^ (Huxley, 1957; Brenner, 1988; Stehle et al., 2002a). For different [P_i_], the statement is correct under the condition that the force per motor remains the same when changing [P_i_]. The latter is supported by several studies on slow (Governali et al., 2020; Smith et al., 2020), fast skeletal (Caremani et al., 2008), and cardiac muscle preparations (Ebus et al., 1994; Zhao and Kawai, 1996).

To implement the [P_i_] dependence of the force and *k*_TR_ associated with P_i_ binding in this model, the (fixed) rate constant *f* ^*–*^ was replaced by an apparent rate constant *f* ^–^_app_ defined by Equation 1:

$$f^{–}\text{app}=f^{–}0+f^{–}+\text{Pi}$$ (1)

where *f* ^–^_0_ is the value of *f* ^–^_app_ at standard [P_i_], i.e., the [P_i_] in the standard activating solution, and *f* ^–^_+Pi_ is an arbitrary function of [P_i_] describing the change in *f* ^–^_app_ from standard [P_i_] to a given [P_i_].

Then, at standard [P_i_]:

$$F_{0}\propto f/\left(f+g+f^{–}0\right)$$ (2a)

$$k_{\text{TR},0}=f+g+f^{–}0$$ (2b)

where *F*_0_ is *F* and *k*_TR,0_ is *k*_TR_ at the standard [P_i_].

At any given [P_i_]:

$$F_{+\text{Pi}}\propto f/\left(f+g+f^{–}0+f^{–}+\text{Pi}\right)$$ (3a)

$$k_{\text{TR},+\text{Pi}}=f+g+f^{–}0+f^{–}+\text{Pi}$$ (3b)

where *F*_+Pi_ is *F*, and *k*_TR,+Pi_ is *k*_TR_ at the given [P_i_], respectively.

Inserting Equation 2b in Equation 2a yields

$$F_{0}\propto f/k_{\text{TR},0}$$ (4a)

Inserting Equation 3b in Equation 3a yields

$$F_{+\text{Pi}}\propto f/k_{\text{TR},+\text{Pi}}$$ (4b)

Dividing Equation 4b by Equation 4a results in

$$F_{+\text{Pi}}/F_{0}\propto \left(f/k_{\text{TR},+\text{Pi}}\right)/\left(f/k_{\text{TR},0}\right)=k_{\text{TR},0}/k_{\text{TR},+\text{Pi}}$$ (5)

Equation 5 demonstrates that the force at the respective [P_i_] normalized to the force at the standard [P_i_] is reciprocally related to *k*_TR_ at the respective [P_i_] normalized to *k*_TR_ at the standard [P_i_]. Therefore, changes in [P_i_] result in reciprocal alterations in force and *k*_TR_.

Equation 5 can be expressed as

$$k_{\text{TR},\text{Pi}}=k_{\text{TR},0}F_{0}/F_{\text{Pi}}$$ (6)

where *k*_TR,Pi_ is *k*_TR_ and *F*_Pi,_ is the force at a given [P_i_]. *k*_TR,0_ is *k*_TR_ and *F*_0_ is the force at basal [P_i_].

The *CS* is defined as 0 when P_i_ alters the force without changing *k*_TR_ (*k*_TR_ = *constant*) and is defined as 1 for maximum coupling as described in Equation 6.

Based on the two edge cases for *CS* = 0, *k*_TR,Pi_ = *k*_TR,0_, and *CS* = +1, *k*_TR,Pi_ = *k*_TR,0_ *F*_0_/*F*_Pi_ (Equation 6), an empirical equation was formulated to describe the intermediate shapes of the P_i_-modulated *k*_TR_-force relation in terms of a linear scale for *CS* within the interval [0, +1]:

$$k_{\text{TR},\text{Pi}}=k_{\text{TR},0}\left(\text{CS}\left(F_{0}/F_{\text{Pi}}\;‐\;1\right)+1\right)=k_{\text{TR},0}\left(1\;‐\;\text{CS}+\text{CS }F_{0}/F_{\text{Pi}}\right)$$ (7)

*CS* can be negative, i.e., *k*_TR,Pi_ can decrease with decreasing *F*_Pi_ if rate-limiting transitions *f* and *f* ^–^ occur after rapid P_i_ release-rebinding. In this case, increasing [P_i_] further reduces the force by shifting the cross-bridges back to non-force-generating states via P_i_ binding. However, *k*_TR,Pi_ decreases because the starting state of the rate-limiting forward transition *f* to force-generating states is the post-P_i_ release state, which is also the P_i_-rebinding state. Increasing [P_i_] lowers the occupancy of this state via P_i_ rebinding and therefore the contribution of *f* for rate modulating *k*_TR_. Consequently, *k*_TR_ decreases from *f* + *f* ^–^ + *g* at low [P_i_] to *f* ^–^ + *g* at high [P_i_]. The empirical equation describing the relation between *k*_obs_ and force due to decreasing *f* (Poggesi et al., 2005) was transformed to describe *k*_TR_-force relations with a negative *CS* in the interval (−1, 0], i.e., for *CS* > −1 and ≤0.

$$k_{\text{TR},\text{Pi}}=k_{\text{TR},0}\left(1+CS\right)/\left(1+CS\;F_{\text{Pi}}/F_{0}\right)$$ (8)

The assumption for deriving (Equation 8) is that basal [P_i_] is zero. This assumption is not required to derive Equation 7, for which any standard [P_i_] can be defined as the basal [P_i_].

**FIGURE 2:**
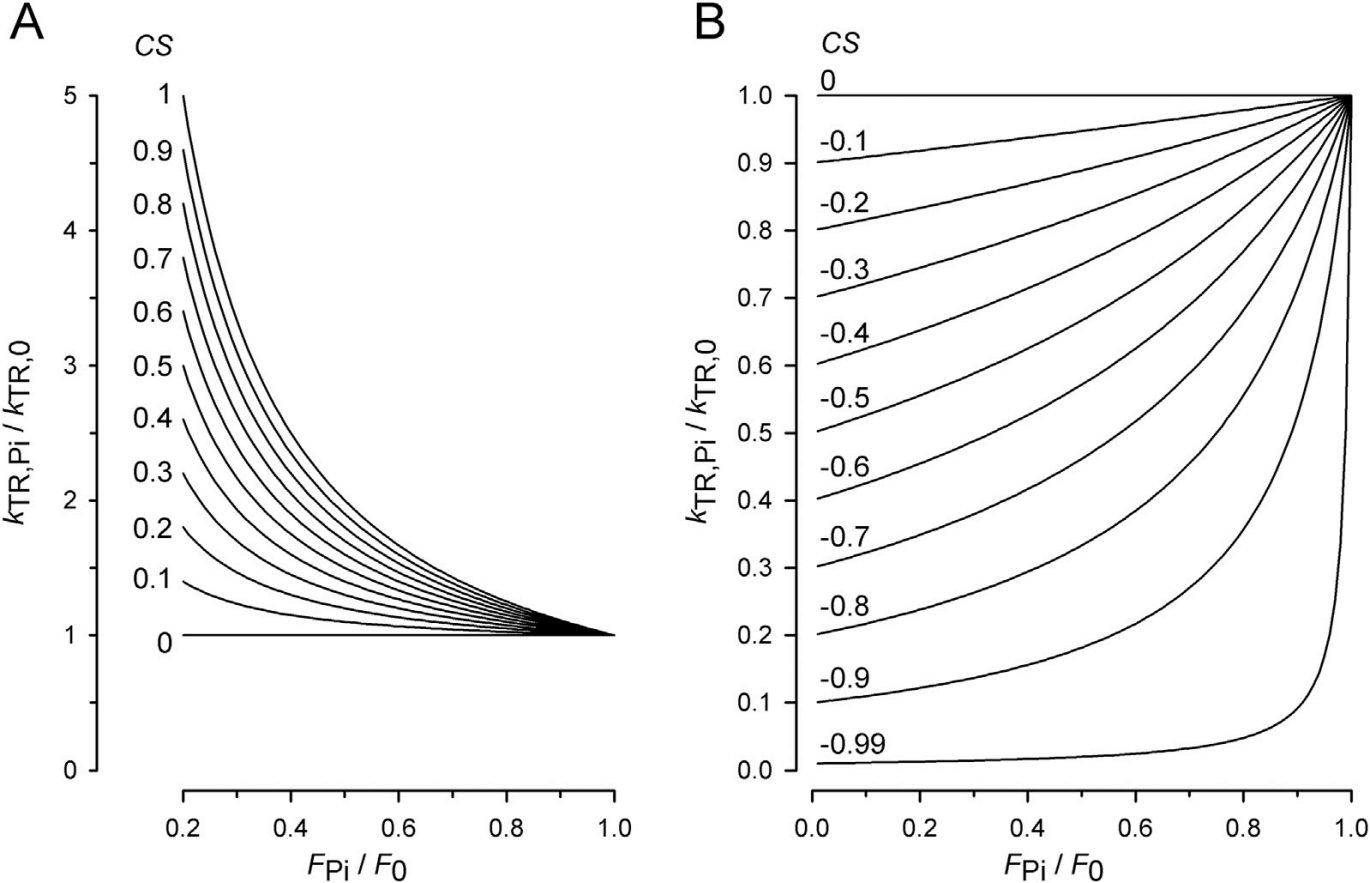
Effect of coupling strength (*CS*) on normalized *k*_TR_–force relations. **(A)** Relations for positive *CS* calculated by Equation 7. Increasing the *CS* by constant step sizes from 0 to 1 results in equidistant series of relations. For *CS* = 1, Equation 7 becomes equivalent to Equation 6 and *y* = 1/*x*. **(B)** Relations for negative *CS* calculated by Equation 8. Decreasing the *CS* by constant step sizes results in equidistant decreases of *k*_TR,Pi_-values at the ordinate. Note that for zero *CS*, *k*_TR,Pi_ = *k*_TR,0_ and *y* = 1 for Equation 7 as well as for Equation 8.

To illustrate the dependence of *k*_TR_-force relations on *CS*, the normalized *k*_TR_ (*y* = *k*_TR,Pi_/*k*_TR,0_) is plotted versus the normalized force (*x* = *F*_Pi_/*F*_0_) for increasing *CS* from 0 to 1 in increments of 0.1, as calculated using Equation 7 (Figure 2A) and for decreasing *CS* from 0 to −0.9 in increments of −0.1 using Equation 8 (Figure 2B). Starting from a flat, linear relation for zero *CS*, *k*_TR_ increases with force reduction by P_i_; *CS* becomes positive. Conversely, the more *k*_TR_ decreases with force reduction, the more *CS* becomes negative. The magnitude of change in *k*_TR_ and the curvature of the *k*_TR_-force relation increase with the absolute value of *CS*.

Equation 7 and Equation 8 can be used to fit the *k*_TR_-force data of muscle preparations obtained at various [P_i_] to derive the *CS* from the experimental data or data obtained by model simulations.

Model simulations were performed using the Berkeley Madonna 8.3.18 differential equation solver. Graphs and fits of experimental and model data were produced by SigmaPlot 8.0. Statistic F-test was performed under GraphPad Prism 4.

## 3 Results

### 3.1 Characteristics of force redevelopment at different [P_i_]

**FIGURE 3:**
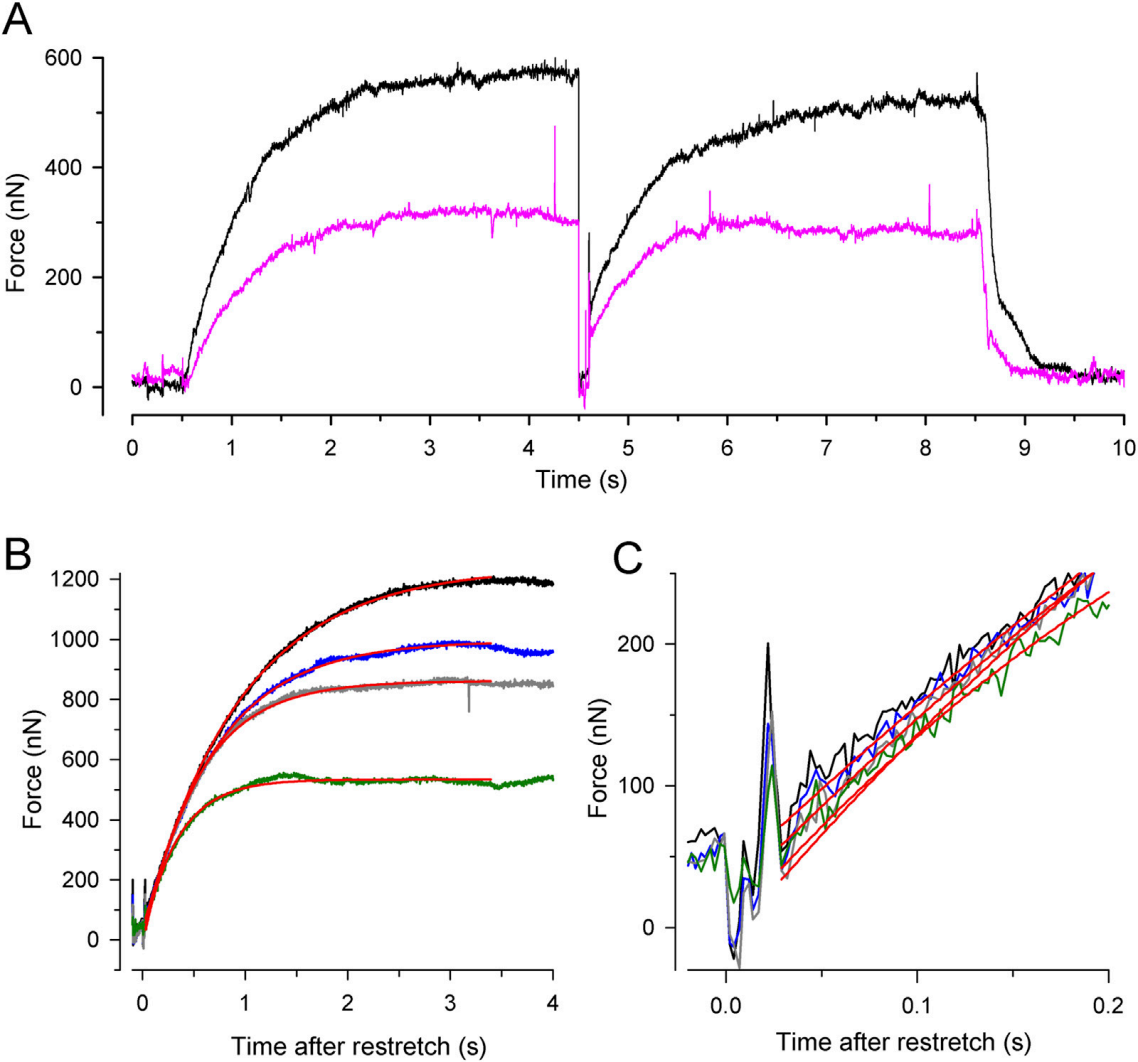
Experimental protocol and force redevelopment at different [P_i_] at 10°C. **(A)** Typical full force transients obtained from a myofibril bundle (2.3 µm diameter, 66 µm length) at 0.17 ± 0.04 mM P_i_ (contaminant [P_i_] in standard buffer, black transient) and 20 mM P_i_ (pink transient). At *t* = 0.5 s, the bundle was activated by switching from relaxing solution (pCa 8) to activating solution (pCa 4.5). At *t* = 4.5 s, the bundle was slackened for 100 ms by 15% of its length and then re-stretched to the original length to induce force redevelopment. At *t* = 8.5 s, the bundle was relaxed by switching back to relaxing solution. Force redevelopment after re-stretch mostly starts from a higher level than slack force like in this example. **(B)** Force redevelopment transients from a myofibril bundle (3.2 µm diameter, 47 µm length) at 1 mM P_i_ (*black*), 2.5 mM P_i_ (*blue*), 10 mM P_i_ (*grey*), and 20 mM P_i_ (*green*). Red lines are single exponentials fitted to transients yielding values for *k*_TR_ of 1.4 s^−1^ (1 mM P_i_), 1.7 s^−1^ (2.5 mM P_i_), 1.9 s^−1^ (10 mM P_i_), and 2.9 s^−1^ (20 mM P_i_). In this experiment, force redevelopments started close to slack force enabling the comparison of their initial force rises that exhibit similar slopes as shown in **(C)**.

Figure 3 shows the force transients of the guinea pig cardiac myofibrils at 10°C, pCa 4.5, and different [P_i_]. The force recordings in Figure 3A illustrate the experimental protocol. The myofibril bundle was exposed to the flow of the relaxing solution (pCa 8), and calcium-induced force development was initiated by rapidly switching to the flow of the activating solution (pCa 4.5), consisting of the same [P_i_] as the relaxing solution. To measure *k*_TR_ during steady-state Ca^2+^ activation, the kinetics of force redevelopment following a transient period of active unloaded shortening was induced (Brenner, 1988). This was performed by applying a slack and re-stretch maneuver to the myofibril bundle consisting of a fast release by 15% of myofibril length to induce unloaded shortening for 50 ms and then a rapid stretch to the original length. After the redevelopment of the force, the bundle was relaxed by switching back from the activating to the relaxing solution. Subsequently, the next activation-*k*_TR_-measurement-relaxation cycle is performed at the next [P_i_].

Increasing [P_i_] reduced the isometric force and the time required to reach the force plateau (Figures 3A, B). Force transients were fitted using single exponential functions (red lines in Figures 3B, C) to determine the rate constant of tension redevelopment, *k*_TR_. Increasing [P_i_] from 1 mM up to 20 mM decreased force by approximately 50% and increased *k*_TR_ by approximately 2.1-fold (Figure 3B), while the initial slope of the force redevelopment remained relatively constant (less than 15% change, Figure 3C). The constant initial slope is expected when the rate constant of an exponential function changes reciprocally with its amplitude, indicating the maximum possible rate modulation of *k*_TR_ (Equation 6).

### 3.2 Dependence of force and *k*_TR_ on the [P_i_]

**FIGURE 4:**
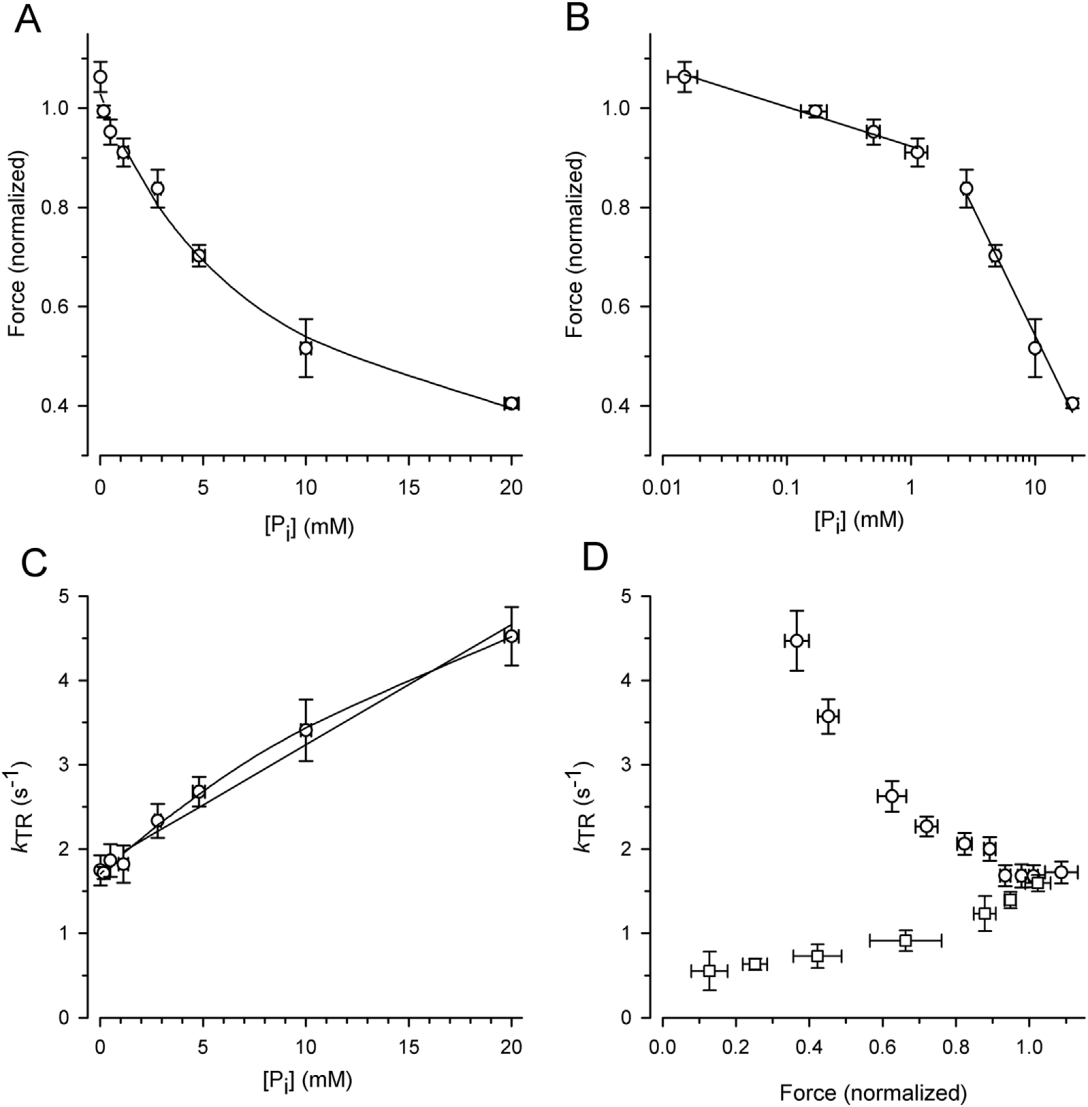
Alteration of force and *k*_TR_ by [P_i_] and comparison of *k*_TR_-force relations resulting from varying [P_i_] and [Ca^2+^] at 10°C. For each myofibril, force data was normalized to force measured in standard activating solution (0.17 mM P_i_, pCa 4.5). **(A)** Force-[P_i_] relation. The line present the hyperbolic function fitted to the data yielding *F*_0Pi_ = 1.03 ± 0.02, *P*_*i*50_ = 8.4 ± 2.1 mM and *F*_∞Pi_ = 0.13 ± 0.09. **(B)** Force-log [P_i_] relation. Lines indicate linear regression lines with slopes of −0.08 per decade increase of [P_i_] at low [P_i_] (≤1 mM P_i_) and −0.51 at high [P_i_] (≥2.5 mM P_i_). **(C)** *k*_TR_-[P_i_] relation and analysis of its curvature. The lines present linear (*k*_0Pi_ = 1.81 ± 0.07 s^-1^, slope = 0.143 ± 0.008 s^-1^/mM P_i_) or hyperbolic fit functions (*k*_0Pi_ = 1.70 ± 0.04 s^−1^, *P*_*i*50_ = 33 ± 9 mM and *k*_∞Pi_ = 9.2 ± 1.3 s^−1^) to the data. **(D)** Relations of *k*_TR_ versus force altered either by changing the [P_i_] between 0.015 mM and 20 mM (*circles*) at full Ca^2+^ activation (pCa 4.5) or by changing the pCa between 4.5 and 5.88 (*squares*) at constant [P_i_] of 0.17 mM. Data was sorted for increasing normalized force values, subdivided in similar groups of n = 14–15 and plotted as mean ± s.d. For force and mean ± SEM for *k*_TR_.

To exploit the force reduction and rate modulation of *k*_TR_ over a broad range of [P_i_], force redevelopment transients were recorded from 19 myofibril bundles at variable [P_i_] ranging from 0.015 mM to 20 mM. The force values of the transients were then normalized to the mean force produced by the myofibril bundle in the standard activating solution containing a contaminant [P_i_] of 0.17 mM. Figure 4A illustrates the relationship between the normalized active force and [P_i_]. Force reduction was already observed at low, sub-millimolar [P_i_] levels. Fitting the force-[P_i_] relation using a hyperbolic function yields three parameters: the fit value at zero [P_i_] (*F*_0Pi_), [P_i_] for half-maximum hyperbolic change (*P*_*i*50_), and the final value at infinity [P_i_] (*F*_∞Pi_). The fitted *F*_∞Pi_ (0.13 ± 0.09) suggests an active force component that cannot be inhibited by P_i_. Plotting force on a logarithmic scale of [P_i_] revealed a bilinear relationship with a 6-fold less steep decrease in force per decade increase of [P_i_] for data with ≤1 mM P_i_ than for the data with ≥2.5 mM P_i_ (Figure 4B).

Figure 4C illustrates the increase in the *k*_TR_-data with the [P_i_], which can be fitted with a linear and hyperbolic function. If a process other than P_i_ binding limits the backward transition from force-generating to non-force-generating states (*f* ^–^), *k*_TR_ saturates at high [P_i_], resulting in a hyperbolic *k*_TR_-[P_i_] relation. In contrast, if *f* ^*–*^ refers to the rate constant of P_i_ binding, a linear increase in *k*_TR_ with [P_i_] is expected. A weak curvature in the *k*_TR_-[P_i_] relation was observed, and the hyperbola fits the data significantly better (*p* = 0.0052 yielded by F-test) than the linear curve (lines in Figure 4C).

The effects of [P_i_] on *k*_TR_ and force were analyzed by plotting the *k*_TR_-force relation, i.e., by pairing the *k*_TR_ values with the corresponding relative force from the same transient (Figure 4D, circles). Because force decreases with increasing *k*_TR_, the *k*_TR_-force relation exhibits a negative slope that becomes steeper at low forces. To determine whether *k*_TR_ simply increased owing to the lower isometric force, the force was reduced by reducing [Ca^2+^] in the standard activating solution without adding P_i_. Force transients from eight myofibrils were recorded at both full and partial Ca^2+^ activation. The force of each transient was normalized to the force at full Ca^2+^ activation (pCa 4.5, 0.17 mM P_i_), and the *k*_TR_ value was paired with the normalized force from the same transient and plotted in Figure 4D (square symbols). Consistent with previous studies, Ca^2+^ modulates *k*_TR_ in the same direction as the force (Brenner, 1988; Sweeney and Stull, 1990; Regnier et al., 1995; Wolff et al., 1995; Edes et al., 2007; Norman et al., 2007; Kreutziger et al., 2008; Papp et al., 2014), which is opposite to the P_i_-modulated *k*_TR_-force relation.

### 3.3 Quantification of coupling strength from [P_i_]-modulated *k*_TR_-force data

**FIGURE 5:**
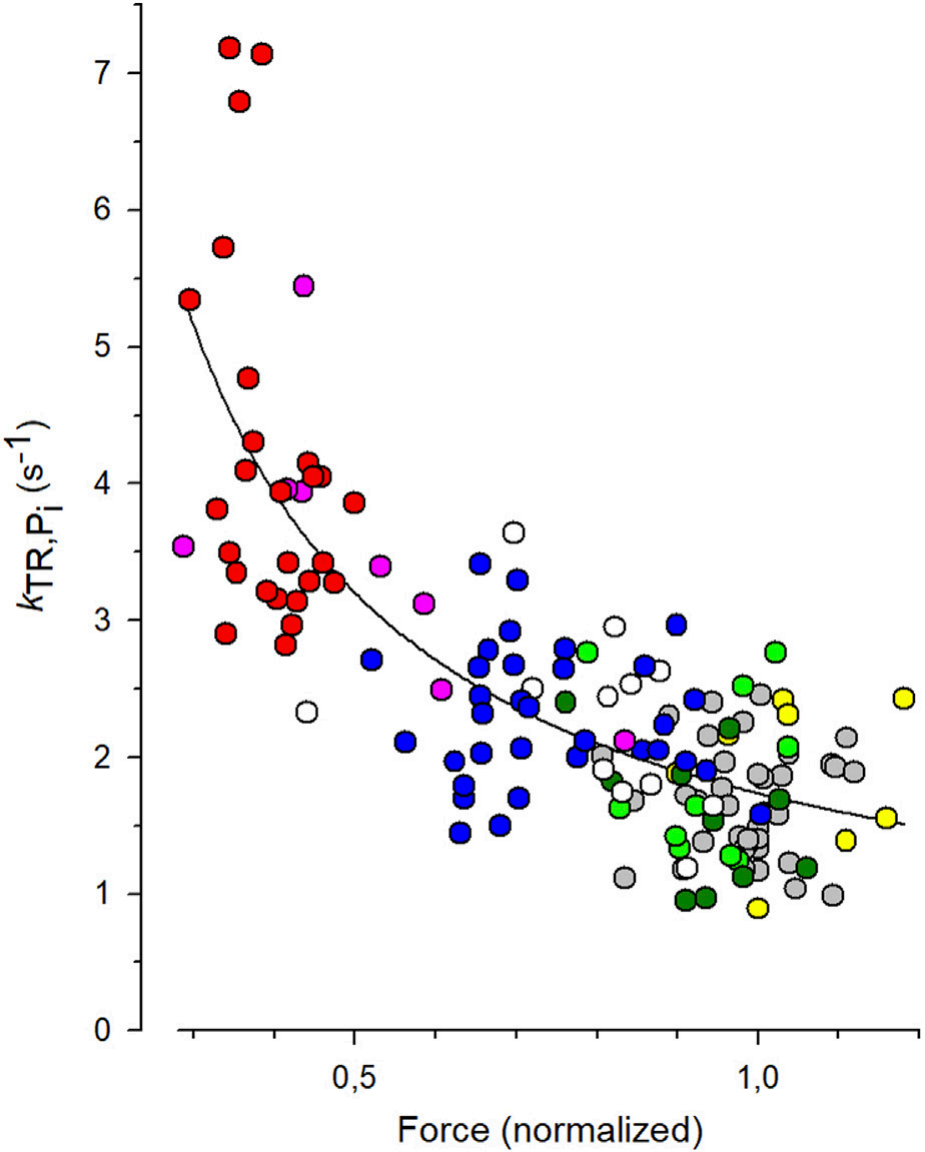
Fit of coupling strength (*CS*) function (Equation 7) to *k*_TR_-force data. All force data is normalized to mean force of each myofibril at 0.17 mM P_i_ that was the contaminant [P_i_] in standard solution. *Grey symbols*: data obtained at 0.17 mM P_i_ (36 transients), *green*: 0.5 mM P_i_ (10 transients), *dark green*: 1 mM P_i_ (10 transients), *white*: 2.5 mM P_i_ (12 transients), *blue*: 5 mM P_i_ (33 transients), *pink*: 10 mM P_i_ (8 transients), *red*: 20 mM P_i_ (26 transients). In three myofibrils, [P_i_] was further reduced by the P_i_ scavenger PNP resulting in a [P_i_] of 0.015 mM (*yellow*, data of 9 transients). The best fit of Equation 7 (line) to the pooled *k*_TR_-force data yields the fit coefficients *CS* = 0.84 ± 0.08 and *k*_TR,0_ = 1.73 ± 0.07 s^−1^ (mean ± s.d).

To quantify the *CS* from the experiments with varying [P_i_], each individual *k*_TR_ value obtained from each transient was paired with the normalized force value from the same transient, and these data pairs were plotted in the *k*_TR,Pi_-force relation shown in Figure 5. The symbols represent the values of 144 force transients obtained from 19 myofibrils at different [P_i_] (indicated by different symbols or colors in the online version). The line represents the best fit of Equation 7 to the data, yielding a *CS* of 0.84 ± 0.08 and *k*_TR,0_ of 1.73 ± 0.07 s^-1^. The latter reflects the *k*_TR_-value of the fit curve at unity force in the standard solution, which is 0.17 mM P_i_.

### 3.4 Rate modulation of *k*_TR_ by [P_i_] and coupling strength depend on the model

**FIGURE 6:**
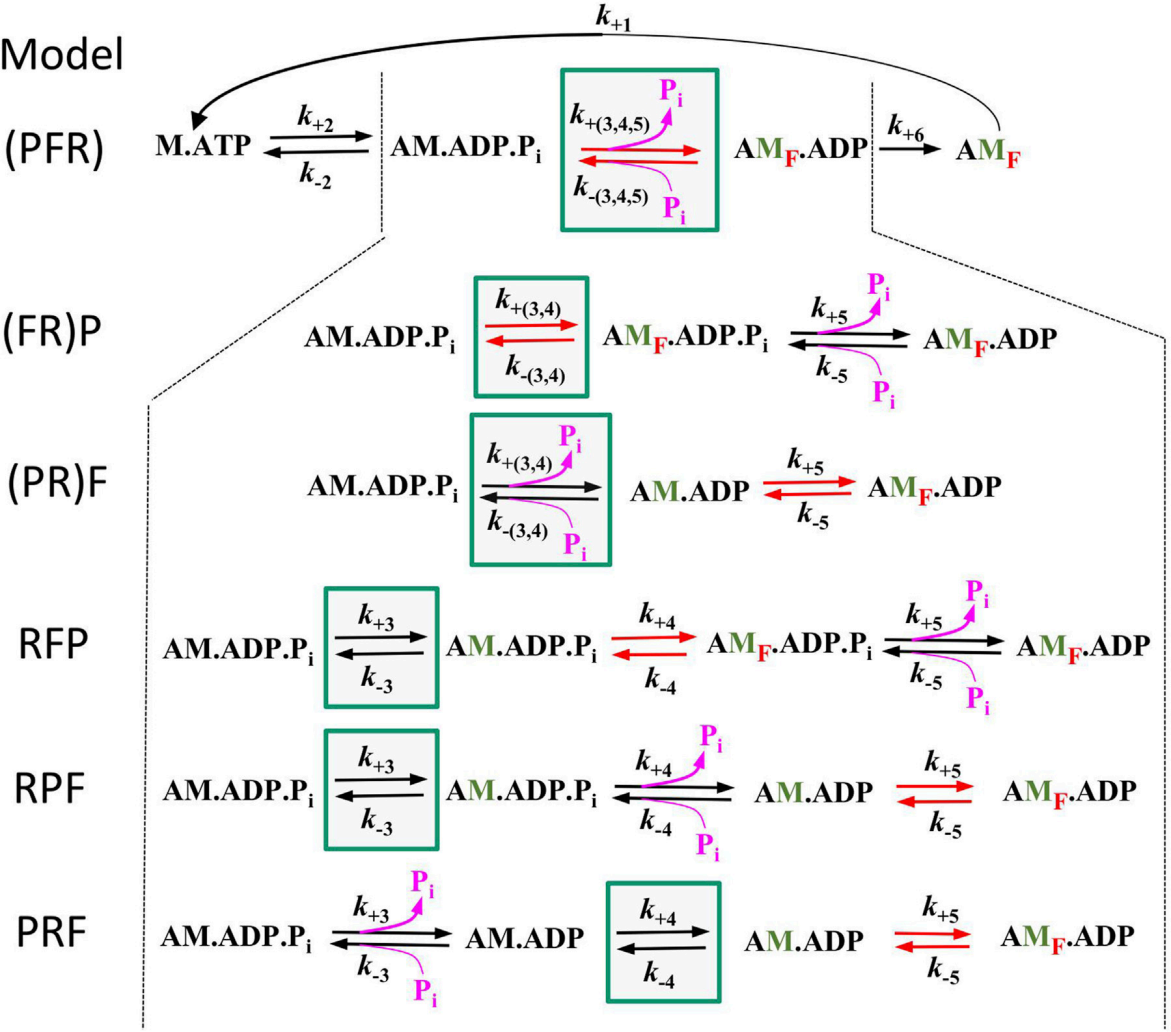
Schemes of the 6 different models tested in this study. All models are equal in step 1 (ATP binding), step 2 (ATP hydrolysis), and step 6 (load dependent forward transition, *g*) but differ in step 3–5. G*reen boxes* mark the equilibrium for rate-limiting forward and backward transitions *f* and *f* ^–^(equilibrium R), *red arrows* mark the equilibrium of the force-generating step and its reversal (equilibrium F) and *pink arrows* the equilibrium of P_i_ release-rebinding (equilibrium P). Lettering of models from left to right indicate the sequence of equilibria in forward direction, brackets indicate inclusion of F or P or both with R. Green labelled ‘M′ indicate strongly bound myosin states that have undergone the rate limiting transition, red subscript ‘F′ in states indicate force-producing myosin states.

To assess the compatibility of the high *CS* obtained in the myofibril experiments with cross-bridge cycle models, various models with different sequences and kinetics for three critical events determining the reversible transition into force-generating states were tested for their [P_i_]-dependent modulation of force and *k*_TR_. Three critical events were defined as reversible equilibria: an equilibrium abbreviated as R for the rate-limiting forward and backward transitions (*f* and *f* ^–^), an equilibrium abbreviated as F for the force-generating step and its reversal, and an equilibrium abbreviated as P for P_i_ release-rebinding. The equilibria R, F, and P were incorporated into various models of the cross-bridge cycle using the same rate constants for ATP binding (step 1), ATP hydrolysis (step 2), and load-dependent ADP release (step 6), but with different sequences of R, F, and P (steps 3–5) and different associations of P or F with R. Models were named by the letters from left to right according their sequence in forward direction of the cycle indicating the sequence of steps for forward transitions. The sequence of steps for backwards transitions results from reading the letters of model names from right to left. Parentheses in names mean that P or F or both are merged with R to single slow equilibrium resulting in combined rate constants (P = R, F = R, and P = F = R), To simulate scenarios where F or P, or both, act as the rate-limiting forward-backward transition, they were combined with R into a single equilibrium, indicated by enclosing either F or P or both with R by a parenthesis in the model name. The different models and their corresponding rate constants are described in Table 1, their schemes are illustrated in Figure 6.

**TABLE 1:** Rate constants for simulations of 6 different models of the cross-bridge cycle.

Model		RFP		RPF		PRF		(PR)F		(FR)P		(PFR)	
Step													
1	*k*_+1_	200											
2	*k*_+2_	10											
	*k*_-2_	2											
3	*k*_+3_	R	1.7	R	1.7	P	8	P = R	1.7	F = R	1.6	P=F=R	1.35
	*k*_-3_		2.5		1.7		1*		0.25*		5		0.15*
4	*k*_+4_	F	17	P	17	R	0.8		n/a		n/a		n/a
	*k*_-4_		25		1.7*		1		n/a		n/a		n/a
5	*k*_+5_	P	17	F	17	F	8	F	17	P	16		n/a
	*k*_-5_		2.5*		17		10		25		1.6*		n/a
6	*k*_+6_	0.5											

Models differ in attribution of step 3–5 (see Figure 6) to the reversible equilibria R, F, and P where R presents the equilibrium of the rate-limiting forward and backward transitions *f* and *f* ^*–*^, F presents the equilibrium of the force-generating step and its reversal, and P presents the equilibrium of P_i_ release-rebinding. Models were named according the sequence of steps, i.e., sequence of letters from left to right indicate the sequence of forward transitions and from right to the left the sequence of backward transitions. Parentheses in names mean that P or F or both are merged with R to single slow equilibrium resulting in combined rate constants (P = R, F = R, and P=F=R), omitted steps (rate constant: n/a) and less steps in that models. Step 1 and step 6 are assumed to be irreversible (reverse rate constants = 0), steps 2–5 are reversible equilibria. Rate constants for step 1 and 2 were derived from stopped flow experiments on cardiac myofibrils from guinea pig (unpublished author own data) using methods described in (Stehle et al., 2000). Rate of step 6 is derived from *k*_TR_, at low [Ca^2+^] shown in Figure 4D. Unit of rate constants is s^-1^ except for the second order rate constant of P_i_ rebinding, *k*_B_ [mM^-1^s^-1^]*. Values of rate constants were selected by following criteria: 1) When P or F are fast equilibria separate from R, their rate constants are 10-fold those of R. 2) The forward rate constant of R was set to match the observed *k*_TR_, at low [P_i_], except for the special case of the PRF, model, were the sum of forward and reverse rate constants of R had to be set to match the *k*_TR_, at low [P_i_]. 3) The reverse rate constants of R, F and P were set to match the force reduction at high [P_i_].

To determine *k*_TR_-values and force for each model, force redevelopment transients were simulated for each model and [P_i_]. This involved the calculation of the steady-state distribution of states during unloaded shortening (with the forward rate constant of step 6 set to *k*’_+6_ = 50 s^-1^) and then switching it at *t* = 0 to a low value for isometric contraction (*k*_+6_ = 0.5 s^-1^). The simulated transients were then fitted using the same type of single exponential function that was used to fit the transients from the myofibril experiments.

**FIGURE 7:**
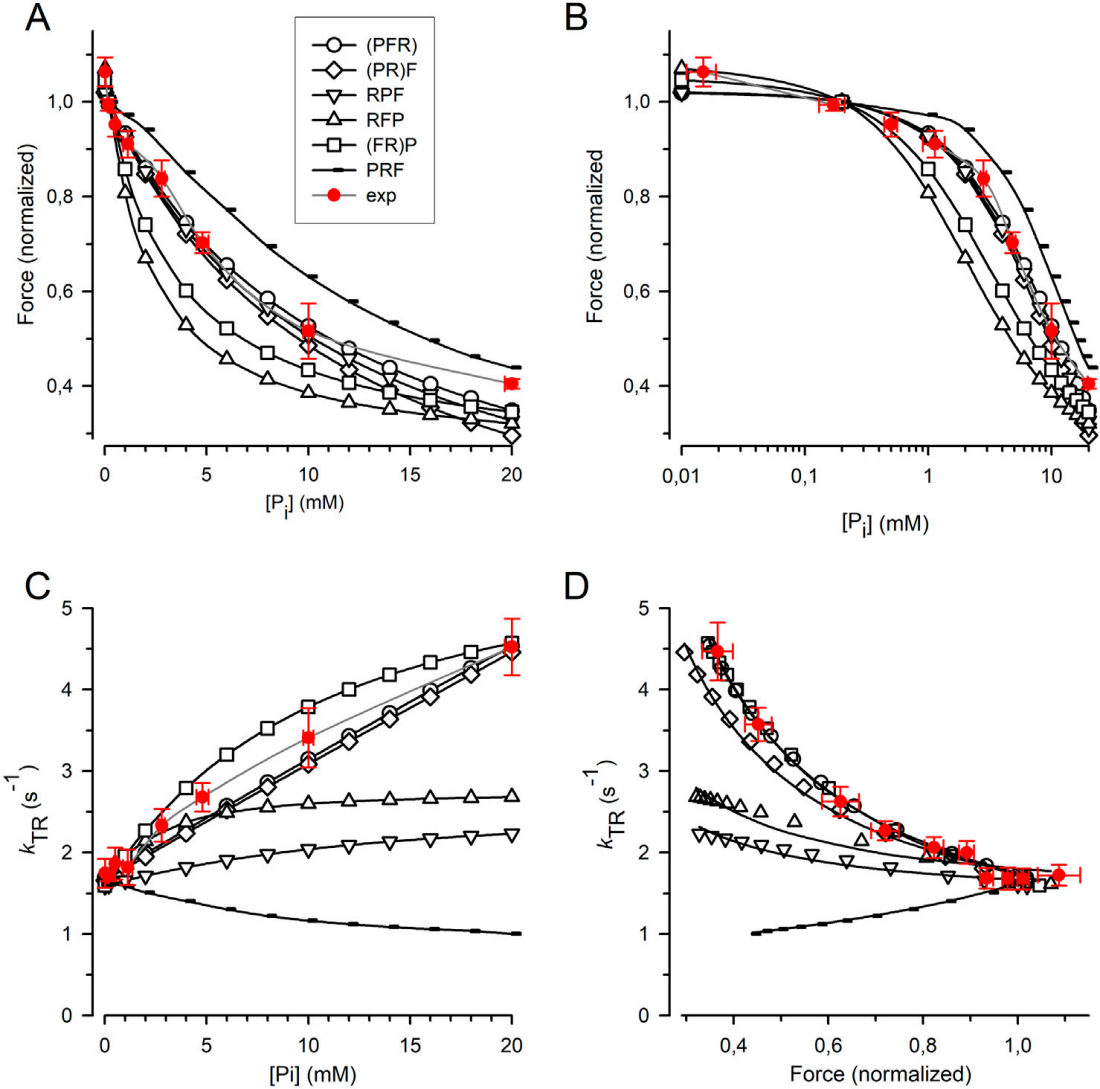
Relation of force and *k*_TR_ on [P_i_] predicted by cross-bridge models differing in sequence and kinetics of reversible equilibria for P_i_ release and force-generating step. **(A)** Force-[P_i_] relations. **(B)** Force-log [P_i_] relations. **(C)** *k*_TR_-[P_i_] relations. **(D)** *k*_TR_-force relations. *Filled red circles* and error bars represent the experimental data replotted from Figure 5. Black symbols indicate relations calculated for the different models: *Open circles* refer to the (PFR) model in which P_i_ release, force-generating step and rate-limiting transition *f* are merged to a single slow step. *Squares* refer to the (FR)P model in which the force-generating step presents the rate-limiting transition *f* followed by faster P_i_ release. *Diamonds* refer to the (PR)F model in which the P_i_ release presents the rate-limiting transition *f* followed by a faster force-generating step. *Tip-up triangles* refer to the RFP-model with the sequence: 1. Rate-limiting transition *f*, 2. Fast force-generating step, 3. Rapid P_i_ release. *Tip-down triangles* refer to the RPF model with the sequence: 1. Rate-limiting transition *f*, 2. Rapid P_i_ release, 3. Fast force-generating step. *Small symbols* present the PRF model with the sequence: 1. Rapid P_i_ release, 2. Rate-limiting transition *f*, 3. Fast force-generating step. Lines in subfigures **(A–C)** are spline curves. Lines in **(D)** represent best fits of Equation 7 to each model except for the RPF model fitted by Equation 8. Model-dependent *CS*: 0.86 ± 0.02 for (PFR), 0.90 ± 0.02 for (FR)P, 0.67 ± 0.02 for (PR)F, 0.26 ± 0.03 for RFP, 0.19 ± 0.03 for RPF, and −0.52 ± 0.01 for PRF. The *CS* of experimental data (*red*) is 0.84 ± 0.08.

The force amplitude for each model was normalized to the force amplitude at 0.17 mM P_i,_ and the normalized force was plotted against either the [P_i_] (Figure 7A) or the log [P_i_] (Figure 7B) together with the relations obtained from the myofibril experiments. The curvature of the myofibril force-[P_i_] relation can be largely described by the (PFR) and (PR)F models, where P_i_ release/rebinding limits the forward/backward transition into/from force-generating states; however, both models overestimate the observed force reduction at the highest [P_i_] of 20 mM P_i_. A similar curvature of the force-[P_i_] relation was predicted by the RPF model, with the sequence of the rate-limiting transition controlling rapid P_i_ release, triggering fast force generation. Models in which force generation precedes rapid P_i_ release predict increased curvatures, regardless of whether F is coupled to R in the (FR)P model or whether F is a fast step following R in the RFP model. The PRF model, in which rapid P_i_ release precedes the rate-limiting transition, yields the lowest curvature. Nevertheless, all the models recapitulate the basic feature of force reduction over a large range of [P_i_], making it difficult to exclude certain models based on the force-[P_i_] and force-log [P_i_] relations.

Figure 7C shows that the *k*_TR_-[P_i_] relation strongly depends on the type of cross-bridge model used. In the (PFR) and (PR)F models, where the backward transition *f* ^–^ is directly limited by P_i_ rebinding, *k*_TR_ increases steeply and linearly with [P_i_]. In addition, a steep but curved increase in *k*_TR_ with [P_i_] was observed when force generation and its reversal were coupled to rate-limiting transitions prior to rapid P_i_ release-rebinding, as in the (FR)P model. The *k*_TR_-[P_i_] relation for cardiac myofibril falls between the linear relations predicted by the (PFR) and (PR)F models and the curved relation predicted by the (FR)P model, which is consistent with these three models. In contrast, the RFP and RPF models in which F and P are fast, reversible equilibria produce less steep *k*_TR_-[P_i_] relations than those observed in the experiments. Notably, the PRF model, in which rapid P_i_ release-rebinding occurs before the rate-limiting transitions *f* and *f* ^−^ predicts a declining *k*_TR_-[P_i_] relation.

To determine the *CS*, Equation 7 (see Methods) was fitted to the *k*_TR_-force data simulated for each model (Figure 7D). Sequential models in which P_i_ release and rebinding were integrated with rate-limiting transitions yielded a high *CS* of 0.86 for the (PFR) model and 0.90 for the (PR)F model) (Figure 7D). The *CS* of these models does not reach the maximum value of 1.0 observed in a two-state model because the ATP cleavage step also participates slightly in limiting the redistribution of cross-bridges between the non-force and force-generating states. The *CS* of both models was in agreement with the *CS* of 0.84 ± 0.08 obtained in the real experiments (Figure 5). A reasonably high *CS* of 0.67 is also observed in the (FR)P model, where fast P_i_ release-rebinding occurred after rate-limiting, reversible force generation. However, when both the force-generating step and P_i_ release are fast, reversible equilibria separate from the rate-limiting transitions *f* and *f* ^–^, the *CS* becomes low, regardless of whether the force-generating step occurs before (RFP model, *CS* = 0.26) or after P_i_ release (RPF model, *CS* = 0.19). The prerequisites for significant rate-modulation of *k*_TR_ and high *CS* is less the sequence of F and P than their kinetics. Finally, inverted rate modulation of *k*_TR_ occurs when reversible P_i_ release precedes the reversible rate-limiting transition as in the PRF model which results in a negative *CS* of −0.51.

## 4 Discussion

### 4.1 Implications of force-[P_i_] and force-log [P_i_] relations for the mechanism of force generation

The asymptote of the hyperbolic fit to the force-[P_i_] relation provides an estimate of the relative active force remaining at infinite [P_i_] (*F*_∞Pi_). *F*_∞Pi_ value <0 indicates that saturating [P_i_] cannot fully reverse the active force, possibly due to the presence of force-producing AM.ADP.P_i_ states (Kawai and Halvorson, 1991; Millar and Homsher, 1992; Tesi et al., 2002) or due to limited reversibility of the process of P_i_ release-associated force generation, i.e., energy dissipation during this process preventing complete reversal of active force. Energy dissipation is usually not reflected by models because they treat cross-bridges as closed system. Because also all the models in this study simulated in Figure 7 assume that the P_i_ release *per se* and force generation is fully reversible, only the force-[P_i_] relations of the RFP and (FR)P models, in which force is generated before P_i_ release, do not approach zero force at infinite [P_i_] (Figure 7A). In all other models, *F*_∞Pi_ = 0. Notably, studies on fast skeletal muscles, such as those performed on skinned fibers (Millar and Homsher, 1990; Pate et al., 1993; Wahr et al., 1997; Wang et al., 2015), exhibited higher asymptote values than those performed on myofibrils (Tesi et al., 2000; Tesi et al., 2002). This discrepancy is attributed to P_i_ accumulation and gradients in fibers, which lead to an underestimation of force modulation by P_i_ (Cooke and Pate, 1985; Cooke et al., 1988), particularly in thicker fiber preparations with high ATPase activity as fast skeletal fibers (Kentish, 1986), emphasizing the need to evaluate *F*_∞Pi_ with cardiac myofibrils.

The value of *F*_∞Pi_ = 0.13 ± 0.09 obtained in this study is consistent with those obtained using fibers (Kentish, 1986), myocytes (Araujo and Walker, 1996; Hinken and McDonald, 2004), and myofibrils (Stehle, 2017) from the cardiac muscle. Interestingly, Tesi et al. explored the force-[P_i_] relation of rabbit psoas and of rabbit soleus myofibrils up to 70 mM P_i_ reporting a similar low asymptote value of 0.07 ± 0.02 for the myofibrils from the fast but a much higher value of 0.44 ± 0.06 for those of the slow muscle which they attributed to a highly occupied force-producing AM.ADP.P_i_ state in slow skeletal muscle (Tesi et al., 2000; Tesi et al., 2002). Also direct comparisons between slow and fast skeletal muscle fibers revealed that slow rabbit soleus fibers exhibit less efficient force reduction than fast rabbit psoas muscle fibers (Millar and Homsher, 1992; Potma et al., 1995; Wahr et al., 1997). Given the low *F*_∞Pi_, the force-[P_i_] relations of myofibrils observed in cardiac and fast skeletal muscles suggest minimal contributions from force-producing AM.ADP.P_i_ state(s) in these muscles. However, due to the limited [P_i_] range of the relations such states cannot be completely excluded. Possible explanations include lower reversibility of the process of P_i_ release-associated force generation or a higher occupancy of the force-producing AM.ADP.P_i_ state in slow skeletal than in fast skeletal or cardiac muscle. This is not simply related to the myosin heavy chain (MHC) isoform since rabbit soleus myofibrils (Tesi et al., 2002) and guinea pig cardiac myofibrils investigated in this study both contain slow β−MHC (Reiser and Kline, 1998). However, it has been recently shown for human β-MHC that differences in the isoform of myosin light chain 1 (MLC1) in slow skeletal and cardiac muscle account for 3-fold lower detachment rates and velocities of actin sliding for slow skeletal compared to cardiac myosin (Osten et al., 2022; Wang et al., 2022). Thus, despite of the same β-MHC, slow skeletal myosin appears to have 3-fold longer attachment times of post-power stroke states than cardiac myosin. Whether this prolonged attachment can result in reduced reversibility of the process of P_i_ release-associated force generation explaining the high residual active force of slow skeletal muscle remains to be tested. Limited reversibility linked to longer attachment times could also provide a protective mechanism for preserving force even at high [P_i_], low pH and elevated [ADP] during muscle fatigue in this muscle type (Karatzaferi et al., 2017; Moretto et al., 2022).

The shape of the force-log [P_i_] relation in skeletal and cardiac muscle preparations and its significance for the force-generating mechanism have been previously described (Pate and Cooke, 1989; Araujo and Walker, 1996; Tesi et al., 2000; Tesi et al., 2002). Based on reduced models consisting of reversible equilibria for the force-generating step and P_i_ release not implemented in a full cross-bridge cycle, a nearly mono-linear force-log [P_i_] relation is expected for the one-step model when force generation and P_i_ release occur simultaneously (Pate and Cooke, 1989), whereas a sigmoidal relation is expected for two-step models, e.g., when force generation precedes or occurs after P_i_ release (Tesi et al., 2000; Tesi et al., 2002). In a previous study on cardiac muscles, mono-linear and sigmoid functions were fitted to force-log [P_i_] data obtained from skinned rat myocytes (Araujo and Walker, 1996). Neither fit accurately matched the experimental data, suggesting that a bilinear fit may provide a better fit to the data of Araujo and Walker. Tesi et al. were the first to use a bi-linear function to analyze their data from rabbit psoas myofibrils at 5°C (Tesi et al., 2000). The slopes of the two lines in their study were −0.07 and −0.40, which are comparable to the −0.08 and −0.51 observed in this study (Figure 4B), indicating a close similarity between force-log [P_i_] relations in fast skeletal and cardiac myofibrils. The force-log [P_i_] relation obtained for skinned rat myocytes was interpreted to have no evidence for the two-step model (Araujo and Walker, 1996) while an earlier report on rat ventricular trabeculae (Kentish, 1986) and the myofibril data from fast skeletal muscle were interpreted to be in rough agreement with the two-step model (Tesi et al., 2000). Force-log [P_i_] relations reported for skinned fibers from rabbit soleus and rabbit psoas muscle were mono-linear with higher slope for the slow soleus muscle (Millar and Homsher, 1992). However, only myofibrils enable the exploration of the force-log [P_i_] relation in the sub-millimolar range because the ATPase activity and long lateral diffusion distance of skinned fibers result in lateral [P_i_] gradients of 1–2 mM P_i_ in skinned fibers (Cooke and Pate, 1985). The slopes of the first line at low [P_i_], up to 1 mM P_i_ for fast skeletal myofibrils (Tesi et al., 2000) and cardiac myofibrils (Figure 4B) are higher than the slope of the sigmoidal relation for a simple two-step model. However, a larger strain distribution of force-generating cross-bridges also increases the slope in this [P_i_] range (Pate et al., 1998). Given the similarity of the force-log [P_i_] relations in fast skeletal and cardiac myofibrils and the different interpretations of force-log [P_i_] relations in previous studies of the two muscle types (Araujo and Walker, 1996; Tesi et al., 2000), the force-log [P_i_] relation alone cannot definitively distinguish between the one-step and two-step models.

Compared with previous studies on force-log [P_i_] relations (Kawai and Halvorson, 1991; Millar and Homsher, 1992; Wahr et al., 1997; Tesi et al., 2002), the use of various full cross-bridge cycle models in this study instead of isolated one- or two-step models without integration in a cycle is a major advancement. Interestingly, regarded over the full [P_i_] range, force-[P_i_], and force-log [P_i_] relations simulated for various full cycle models were comparable (Figures 7A, B). At low [P_i_], the results from the full cycle models in this study were opposite to predictions made by the “isolated step” models (Pate and Cooke, 1989; Tesi et al., 2000). While isolated one-step models predict overall mono-linear force-log [P_i_] relation with higher slopes at low [P_i_] than isolated two-step models (Pate and Cooke, 1989; Tesi et al., 2000), in this study, the (PFR) model which integrates the one-step model in the cycle even yields a flatter force-log [P_i_] relation than the RFP model which integrates the two-step model. None of the models explored in this study exhibited a monophasic relation expected from isolated one-step models of P_i_ release-associated force generation. Overall, the simulations with various full-cycle models suggests that the overall shape of the force-log [P_i_] relation is rather insensitive to the specific coupling mechanism between force generation and P_i_ release. Instead, the slope of the force-log [P_i_] relation at low [P_i_] is influenced by the coupling mechanism in a more complex manner than previously understood.

### 4.2 Implications of the k_TR_-[P_i_] relation for the mechanism of force-generation

The addition of 10 mM P_i_ increases *k*_TR_ 2.4-fold in skinned cardiac myocytes from human donor hearts (Papp et al., 2014), which is comparable to the 2.0-fold increase in *k*_TR_ by 10 mM P_i_ observed in cardiac myofibrils from guinea pigs in this study. In rat skinned cardiac myocytes, the addition of 10 mM P_i_ increased *k*_TR_ by 3.8-fold (Hinken and McDonald, 2004), whereas in cardiomyocytes from humans, pigs, and mice, the increases are 1.5-fold, 1,6-fold and 2.9-fold, respectively (Edes et al., 2007). The stronger P_i_ effects in mice and rats compared to those in human, pig, and guinea pig hearts may be partly related to the fast α-MHC isoform present in murine and rat ventricles compared to the slow β-MHC isoform expressed in humans, pigs, and guinea pigs (Reiser and Kline, 1998). However, MHC isoform differences are not the only determinant of the *k*_TR_-[P_i_] relation, since myofibril and fiber preparations from slow skeletal muscle exhibit no change in *k*_TR_ (Wahr et al., 1997; Tesi et al., 2000; Tesi et al., 2002), with one exception where force development kinetics was induced by T-jumps (Governali et al., 2020). In most studies on slow skeletal muscle preparations, the insensitivity of *k*_TR_ to P_i_ could indicate incomplete reversibility within the process of P_i_ release-associated force generation or different force-generating mechanisms in this muscle type (Stehle and Tesi, 2017).

The model simulations in this study revealed that the slope and curvature of the *k*_TR_-[P_i_] relation are sensitive to the force-generating mechanism. The slope is strongly positive for models in which P_i_ binding induced force reversal is slow and limits *f* ^–^. In contrast, the slope is flat for models in which P_i_ binding induced force reversal is a fast process. The slope becomes negative for models in which P_i_ release-rebinding is a fast equilibrium before the rate-limiting transitions *f* and *f* ^–^ in the cycle. The *k*_TR_-[P_i_] relation is linear when P_i_ rebinding directly limits the backwards transition of cross-bridges from force-generating to non-force-generating states, and the relation curves downward when P_i_ rebinding is fast; therefore, another process becomes rate-limiting for this transition at high [P_i_]. Despite the clear prediction of the mechanism, the curvature of the *k*_TR_–[P_i_] relations reported in the literature does not provide a unique picture of the force-generating mechanism. Downward-curved *k*_TR_-[P_i_] relations were reported for skinned fast (Regnier et al., 1995; Wahr et al., 1997) and slow (Wahr et al., 1997; Governali et al., 2020) muscle fibers, a slightly downward-curved relation for myofibrils from rabbit psoas (Tesi et al., 2000), apparently linear *k*_TR_-[P_i_] relation in a previous study of cardiac myofibrils from guinea pig (Stehle, 2017), and even an upward curvature for skinned rat cardiac myocytes (Hinken and McDonald, 2004). The slightly downward curved *k*_TR_-[P_i_] relation obtained for cardiac myofibrils in this study can better fitted by a hyperbola than by a linear function (Figure 4C) suggesting that another process than P_i_ rebinding limits *f* ^–^ at infinite [P_i_]. However, the low curvature represented by the high *P*_*i*50_ = 33 ± 9 mM indicates that at physiological [P_i_], i.e., at [P_i_] up to 30 mM (Cady et al., 1989), the rate of P_i_ rebinding limits *f* ^–^. The high *P*_*i*50_–value and the variability of curvatures of *k*_TR_-[P_i_] relations in literature corroborate the difficulty in determining the force-generating mechanism from the shape of this relation. Therefore, an alternative criterion was explored in this study: the combined modulation of *k*_TR_ and force by [P_i_], i.e., the *k*_TR_-force relation was described in terms of the *CS*.

### 4.3 Implications of the *k*_TR_-force relation for the mechanism of force generation

Early models of the cross-bridge cycle implied that the P_i_ release step is connected to steps that limit the transition of cross-bridges from non-force-generating to force-generating states (Huxley, 1957; Lymn and Taylor, 1971; Eisenberg et al., 1980). In contrast, newer models propose that P_i_ release occurs rapidly, independent of slower step(s) in the ATPase cycle (Millar and Homsher, 1990; Kawai and Halvorson, 1991; Dantzig et al., 1992; Ranatunga, 1999). The latest models involve branched pathways and multiple steps of force generation and P_i_ release (introduction and reviews (Debold, 2021; Mansson et al., 2023)), making it increasingly difficult to identify specific rate-limiting transitions to certain steps in the cycle. Novel insights into the structural cycle of myosin (Muretta et al., 2015; Houdusse and Sweeney, 2016; Irving, 2017; Robert-Paganin et al., 2020; Matusovsky et al., 2021), highly time-resolved force measurements of single myosin (Woody et al., 2019; Scott et al., 2021), and load-dependent, organized conformational changes of the cross-bridge ensemble on the thick filament (Linari et al., 2015; Irving, 2017; Brunello et al., 2020; Park-Holohan et al., 2021) further revive the question of which steps limit forward and backward cycling between non-force-generating and force-generating states.

The first attempt to distinguish P_i_ release-associated force generation from the rate-limiting transition in the traditional, sequential pathway of the cross-bridge cycle was based on classical experiments using caged-P_i_. The rapid increase in [P_i_] produced by the flash photolysis of caged-P_i_ in muscle fibers induces a fast force decay with a rate constant *k*_Pi_ considerably higher than *k*_TR_ (Millar and Homsher, 1990; Dantzig et al., 1992). The dependence of *k*_Pi_ on [P_i_] was interpreted as fast reversible force generation followed by rapid reversible P_i_ release (Millar and Homsher, 1990; Dantzig et al., 1992) reviewed in Takagi et al. (2004): Rapid P_i_ binding and fast force reversal determine the high *k*_Pi,_ while slower transitions limit redistribution among force-generating and non-force-generating states and determine the low *k*_TR_. However, this scenario has been questioned by studies exploring the force kinetics upon rapid changes in [P_i_] in myofibrils (Tesi et al., 2000; Stehle, 2017) reviewed in Stehle and Tesi (2017). Thin myofibril bundles are ideal for studying the force kinetics induced by rapid switching between two solutions of different [P_i_], enabling a change in [P_i_] in both directions, e.g., from initial low [P_i_] or initial high [P_i_] to the same final [P_i_]. Notably, this was first reported for myofibrils from fast skeletal muscle (Tesi et al., 2000) and later for cardiac myofibrils (Stehle, 2017), the force kinetics at the same final [P_i_] are strikingly different depending on the direction of [P_i_] change. Rapid increases in [P_i_] induce fast force decay, as in fibers, whereas rapid decreases in [P_i_] induce slow force rises with *k*_-Pi_ similar to *k*_TR_. Furthermore, in cardiac myofibrils, the fast kinetics of force decay with a rapid increase in [P_i_] was attributed to the sequential “give” of sarcomeres (Stehle, 2017), a phenomenon also observed during fast muscle relaxation (Huxley and Simmons, 1970; Flitney and Hirst, 1978; Stehle et al., 2002a). The rapid decrease in [P_i_] to a low final [P_i_], i.e., the rapid prevention of backward cycling via P_i_ rebinding, induces forward kinetics limited by the same transition limiting force redevelopment, implying that P_i_ release coupled force generation in the forward direction is linked to the rate-limiting transition *f* (Stehle and Tesi, 2017).

This study was the first to analyze the *CS* between the processes of P_i_ binding induced force reversal and transition limiting backward cycling, represented by the rate constant *f* ^–^. Empirical equations were developed to derive this *CS* from *k*_TR_-force relations. The major assumption for deriving Equations 7, 8 for describing *CS* is that the rate constant of force redevelopment represents the sum of the rate constants limiting redistribution between the non-force- and force-generating states. However, this assumption has been challenged by Kawai, who argued that cross-bridges must complete multiple cycles because their step size is much smaller than the distance of the filament sliding during force redevelopment (Wang and Kawai, 2013). A prediction of Kawai’s model (Wang and Kawai, 2013) is that *k*_TR_ is inversely related to the tension cost, defined as the ratio of isometric tension to ATPase. Tension cost is independent of Ca^2+^ activation (Brenner, 1988) and increases approximately 2-fold with increasing [P_i_] to 30 mM in fast skeletal and cardiac muscle (Ebus et al., 1994; Potma et al., 1995; Potma and Stienen, 1996; Governali et al., 2020). However, this 2-fold change in tension cost is insufficient to explain the 10-fold or 15-fold difference observed in *k*_TR_ when the force was reduced by altering Ca^2+^ and Pi in cardiac myofibrils (Figure 2D) or fast skeletal muscle fibers (Regnier et al., 1995), respectively. The classical interpretation of *k*_TR_ provides a simple explanation for these substantial differences and opposing changes in *k*_TR_ resulting from force reduction via Ca^2+^ and P_i_ by decreasing *f* and increasing *f* ^–^, respectively (reviewed in (Stehle and Tesi, 2017).

Model simulations reveal that *CS* is high when either the reversible equilibrium for P_i_ release P, the reversible equilibrium for force generation F, or both equilibria are connected to the rate-limiting forward-backward transition between non-force-generating and force-generating states R, as observed in the (PR)F, (FR)P, and (PFR) models. Conversely, *CS* decreases when both F and P are separated from R, as in the RFP and RPF models (Figure 7D). On a scale from +1 for maximum positive over zero for no coupling to −1 for maximum inverse coupling, the *k*_TR_-force relation in cardiac myofibrils yielded a high *CS* of 0.84 close to 1, which is consistent with the (PR)F, (FR)P, and (PFR) models, but not with the RFP and RPF models.

Owing to the linear scaling of the positive *CS* defined by Equation 7 (illustrated in Figure 2A), positive *CS* reflects the ratio of the relative increase in *k*_TR_ to the relative decrease in the force induced by a certain increase in [P_i_]. Therefore, the *CS* of 0.84 in this study indicates that, on average, *k*_TR_ increases by 0.84 times the reduction in force; for example, *k*_TR_ increases 0.84 × 2-fold = 1.68-fold when force is reduced 2-fold to half of its initial value. To the author´s knowledge, no previous study has considered this ratio, while numerous studies on several muscle types have reported *k*_TR_ and force values that contain this information. In skinned rat cardiac myocytes, the addition of 10 mM P_i_ increased *k*_TR_ by 3.8-fold, whereas it reduced the force by 3-fold (Hinken and McDonald, 2004). Similarly, in skinned cardiac myocytes from human donor hearts, the addition of 10 mM P_i_ resulted in a 2.4-fold increase in *k*_TR_ and a 2.5-fold reduction in force (Papp et al., 2014). These changes in *k*_TR_ and force are comparable to the 2.0-fold increase in *k*_TR_ and 2.0-fold reduction in force observed in cardiac myofibrils from guinea pigs, likely because of the similar β-MHC isoform present in guinea pigs and human hearts. However, in all these studies, including those on fast skeletal muscle, *k*_TR_ changes almost reciprocally with force. In fast muscle fibers, the addition of ≥10 mM P_i_ reduced the force by half and doubled *k*_TR_ (Wahr et al., 1997; Linari et al., 2010), whereas the addition of 5 mM P_i_ was sufficient to halve the force and double *k*_TR_ in myofibrils of this muscle type (Tesi et al., 2000; Tesi et al., 2002). Notably, theoretical modeling of [P_i_]-dependent changes in *k*_TR_ and force in fast muscles also displayed sensitive, reciprocal changes in *k*_TR_ and force (Linari et al., 2010). In the model of Linari et al., the formation of strongly bound cross-bridges and force generation were combined with the kinetics of a slow process analogous to the (FR)P model simulated here. Therefore, both experimental and theoretical data from cardiac and fast skeletal muscles support the view that *CS* is high, close to one. However, the *CS* of the slow skeletal muscles remains unclear. The insensitivity of *k*_TR_ to [P_i_] reported for fibers (Wahr et al., 1997) and myofibrils (Tesi et al., 2002) from slow rabbit soleus muscle indicated a low *CS*, even when considering the lower effects of P_i_ on force in slow skeletal muscle than in fast skeletal muscle. However, a recent study on slow rabbit soleus muscle fibers reported sensitive changes in the rate constant of force development kinetics following T-jumps and force by [P_i_] (Governali et al., 2020). Assuming that the latter rate constant reports the same transitions in the cross-bridge cycle as *k*_TR_, the experiments of Governali et al. on slow muscle indicate a high *CS* similar to that observed in fast skeletal and cardiac muscle.

The high *CS* observed in fast skeletal and cardiac muscle may be due to either slow rebinding of P_i_, i.e., low *k*_B_ as in (PFR) and (PR)F models, or slow reversal of the power stroke, as in (RFP) and (FR)P models. A low *k*_B_ does not necessarily contradict the classical prediction of ultra-rapid rate constants from diffusion-limited reactions because P_i_ is released and potentially rebounds through so-called backdoor mechanisms, which may limit the rate of P_i_ release and rebinding (Yount et al., 1995; Cecchini et al., 2010; Houdusse and Sweeney, 2016; Moretto et al., 2022). However, model simulations also revealed a high *CS* for the (FR)P model, in which force generation is coupled to rate-limiting transitions prior to rapid reversible P_i_ release; i.e., a high *CS per se* does not exclude rapid P_i_ rebinding. In the (FR)P model, the rate-limiting backward transition to non-force-generating states, represented by *f* ^–^ is limited by the reversal of the force-generating steps. Force-reverse steps have been detected and quantified using the laser trap technique for single cardiac myosin and cardiac myosin filaments (Woody et al., 2019; Hwang et al., 2021). In the single myosin force experiments conducted by Hwang, two reverse steps were identified in the presence of ADP and P_i_ but in the absence of ATP. The authors, therefore, attributed these two reverse strokes in their model analysis to different actin-bound myosin.ADP states occurring after P_i_ release. However, simply assigning a sequence of reversible power strokes, i.e., forward steps in total to *f* and reverse steps in total to *f* ^–^ after rapid P_i_ release-rebinding in a sequential model would yield a negative *CS* similar to the PRF model. Whenever the post-P_i_ release state precedes the rate-limiting transition into force-generating states, the occupancy of this post-P_i_ release state decreases with increasing [P_i_], whereby *k*_TR_ no longer increases but decreases with [P_i_], i.e., leading to an inverse rate modulation of *k*_TR_ by P_i_. Therefore, to implement a sequence of rate-limiting reverse steps in the full ATPase cross-bridge cycle with a high positive *CS* and rate modulation of *k*_TR_, they must be closely assigned either along or before P_i_ release-rebinding, as seen in the (PFR) or (FR)P models. The scenario involving rapid reversible power strokes before fast reversible P_i_ release aligns with the proposal of Woody et al. (2019). However, the coupling of the power stroke to the rate-limiting transition remains unsolved.

**FIGURE 8:**
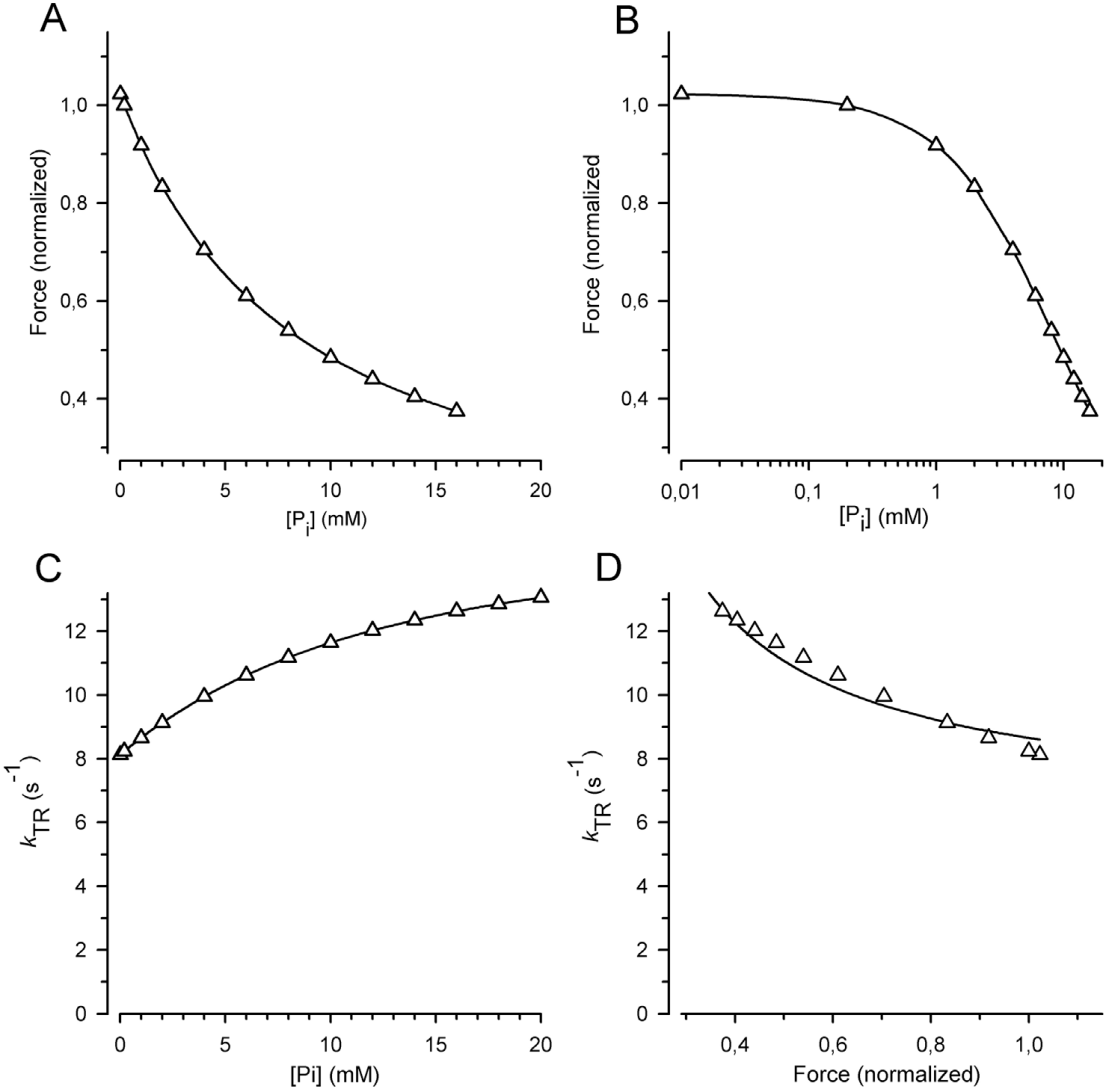
Relation of force and *k*_TR_ on [P_i_] predicted for the classical two-step mechanism of fast force generation followed by rapid P_i_ release in rabbit psoas muscle fibers at 10°C (Millar and Homsher, 1990; Dantzig et al., 1992). **(A)** Force-[P_i_] relations. **(B)** Force-log [P_i_] relations. **(C)** *k*_TR_-[P_i_] relations. **(D)** *k*_TR_-force relations. Relations were calculated using the six-state RFP-type model in which the reversible rate-limiting transition into force-generating states (step 3) controls fast reversible force generation (step 4) that triggers rapid reversible P_i_ release (step 5). Step 1 = ATP binding, step 2 = reversible ATP hydrolysis and step 6 = rate-limiting forward transition for leaving force-generating states (slow isomerization and ADP release). Rate constants of step 1 (*k*_+1_ = 250 s^−1^), and step 2 (*k*_+2_ = 20 s^−1^, *k*_-2_ = 3 s^−1^) were derived from stopped flow experiments with rabbit psoas myofibrils (Herrmann et al., 1992; Stehle et al., 2000). Rate constants of step 3 (*k*_+3_ = 10 s^−1^, *k*_-3_ = 10 s^−1^) and step 6 (*k*_+6_ = 2 s^−1^) were derived from values in the literature for *k*_TR_ (Tesi et al., 2000) and ATPase (Potma et al., 1995). Rate constants of step 4 (*k*_+4_ = 21 s^−1^, *k*_-4_ = 100 s^−1^) and step 5 (*k*_+5_ = 500 s^−1^, *k*_-5_ = 8 s^−1^) are from caged-P_i_ experiments (Dantzig et al., 1992). Lines in subfigures **(A–C)** are spline curves. Lines in **(D)** represent the best fit of Equation 7 to the model data yielding *CS* = 0.28 ± 0.02 (compared to the *CS* obtained by the experiments in this study of 0.84 ± 0.08).

The observed high *CS* contradicts the classical two-step mechanisms of force generation. Traditional sequential pathways involve an intermediate fast reversible force-generating step followed by rapid reversible P_i_ release, with a slower process that rate limits forward and backward fluxes of cross-bridges, i.e., *f* and *f* ^–^ independent of the force-generating step in the cycle (Millar and Homsher, 1990; Kawai and Halvorson, 1991; Dantzig et al., 1992; Ranatunga, 1999; Takagi et al., 2004). This scenario is reflected by the RFP model, which yields a *CS* of 0.26, significantly lower than the *CS* of 0.84 observed in the cardiac myofibril experiments (Figure 7D). Using the rate constants from classical studies favoring the RFP model for rabbit psoas muscle at 10°C (Millar and Homsher, 1990; Dantzig et al., 1992) resulted in a low *CS* of 0.28 (Figure 8). *CS* further decreased to 0.19, when the sequence of the two fast reversible equilibria was permuted and force generation occurred after P_i_ release, as shown by the RPF model in Figure 7D.

High *CS* can be obtained by increasing the rate constants of P_i_ release and force generation in the forward direction, but not in the backward direction. For example, P_i_ release can be made fast in the RFP model (high *k*_P_) when P_i_ binding is slow; i.e., the low *k*_B_ limits the rate of *f* ^–^. The loss of force modulation by [P_i_] resulting from the high *k*_P_/*k*_B_ ratio, i.e., the higher equilibrium constant of the reversible P_i_ release (*K*_P_), must be compensated by lowering the equilibrium constant of the preceding fast reversible force-generating step (*K*_F_). Thereby, it is possible to obtain the rate modulation of *k*_TR_ by [P_i_] by slow rate constant for P_i_ rebinding even for high rate constants of P_i_ release. The combination of low *K*_F_ and high *K*_P_ results in a low occupancy of the post-power-stroke, force-producing AM_F_.ADP.P_i_ state. Because this state is the initial state of P_i_ release, its low occupancy limits the rate of P_i_ release, even though *k*_P_ is high. Thereby, flux to force-generating states is still limited by transition through the P_i_ release-rebinding equilibrium. Nevertheless, to obtain a high *CS*, it is essential to keep either the kinetics of P_i_ rebinding or force reversal slow and coupled to *f* ^–^.

The above combination of low *K*_F_ and high *K*_P_ in the RFP model would result in high free energy for the force-producing AM_F_.ADP.P_i_ state with a large drop in free energy during P_i_ release and high energy barrier for P_i_ rebinding. This could be a problem for efficiency and reversibility of P_i_ release-associated force generation. Given that P_i_ release and rebinding are multiple equilibria (Llinas et al., 2015; Moretto et al., 2022), the energy associated with P_i_ release can be partitioned to more than one step, facilitating P_i_ rebinding and its modulation by pH during muscle fatigue (Moretto et al., 2022). During the multistep P_i_ release reported by Muretto et al., the power stroke occurs in different myosin states after the P_i_ release from the active site. It occurs either while P_i_ is bound to the secondary P_i_ binding site, bound to the surface of myosin or already released free in solution. Whether in such scenario the different rates of P_i_ rebinding could be directly involved in limiting the backward flux of cross-bridges from force-generating states to non-force-generating states needs to be tested. As discussed in 4.2., P_i_ binding limiting *f* ^–^would manifest in linear *k*_TR_–[P_i_] relations as observed in the (PFR) and (PR)F models, and literature reports varying curvatures in *k*_TR_–[P_i_] relations, generally exhibiting slightly downward curvatures, but without evidence of *k*_TR_ approaching a maximum, [P_i_]-independent value at high [P_i_]. Therefore, it is likely that the rate of backward cycling of cross bridges is substantially limited by the rate of P_i_ rebinding, which has not yet been directly measured.

A major limitation of the current model analysis was the simplification to a single sequential pathway. The aim was to outline the primary pathway by comparing various scenarios, rather than increasing the level of complexity or refining a certain model. Owing to the single pathway and full reversibility of the steps in all models, each model predicts a parallel decrease in force and ATPase by P_i_, i.e., [P_i_]-independent ATPase/force ratios, called the tension cost, and cannot account for the well-known observed increase in tension cost with increasing [P_i_]. Studies on skinned cardiac trabecular fibers from swine containing also the slow β-MHC as expressed in the guinea pig indicate that 20 mM P_i_ doubles the tension cost (Herzig et al., 1981; Strauss et al., 1994). Similar increase in tension cost have been found in fast (Potma and Stienen, 1996) and slow skeletal muscle fibers (Governali et al., 2020) leading to models with side pathways to uncouple (Linari et al., 2010) or loosely couple (Caremani et al., 2013; Governali et al., 2020) P_i_ release and force generation. As shown in Figure 1 of Linari et al.’s Study (Linari et al., 2010), adding a non-force-producing side pathway for the uncoupling of ATPase activity from force generation results in a substantial reduction in the [P_i_]-dependence of ATPase by approximately 3-fold. However, this modification produced only minor changes of 15% in the [P_i_] dependences of force and *k*_TR_, with these changes occurring almost reciprocally, indicating that adding a non-force-producing pathway does not significantly alter *CS*. However, cross-bridge models with multiple reversible force-generating steps and loose coupling to P_i_ release (Caremani et al., 2013; Governali et al., 2020) can predict high *CS* even for rapid P_i_ release-rebinding kinetics when the total flux through these steps passes over the P_i_ release-rebinding step and participates in the rate-limiting *f* and *f* ^–^. This is evident from the [P_i_]-dependent reciprocal changes in force and *k*_TR_ simulated for the loose coupling model depicted in Figure 4 of Governali et al. (2020) that yielded a high *CS* close to 1.

The ultrafast, high-sensitive force recordings of a single myosin indicate that myosin generates a power stroke within less than 1 ms upon strong binding to the actin filament (Capitanio et al., 2012). Recent studies using the same technique (Woody et al., 2019; Scott et al., 2021) detected no effects of P_i_ on the attachment time of heads prior to execution or on the size or rate of the initial power stroke. Rapid reversible P_i_ release-rebinding was attributed to the post-power-stroke state, as reflected by a load and [P_i_]-dependent drop in force manifested in the averaged single myosin force transients (Woody et al., 2019). Recent time- and structurally resolved measurements that classify attachment-detachment events in HMM in combination with myosin lever arm orientation also detected no effect of P_i_ on the lifetime between the attachment of the myosin head to actin and the power stroke (Matusovsky et al., 2021). Thus, time-resolved single myosin force and structure studies indicate that P_i_ does not affect the lifetime of the pre-force-generating state. These findings have been interpreted to indicate that strong myosin attachment triggers the power stroke before P_i_ release (Woody et al., 2019; Matusovsky et al., 2021; Scott et al., 2021). However, this interpretation has been challenged by the finding of a secondary P_i_ binding site outside of the active site and the successful modelling of both the [P_i_]-insensitivity of single myosin force transients and the [P_i_]-sensitivity of muscle force transients by a model in which the power stroke occurs after P_i_ release from the active site (Moretto et al., 2022) using multi-scale model simulations (Rahman et al., 2018). Furthermore, there is dissent about the interpretation of the increased amount of short-lived attachments of single cardiac myosin found at high [P_i_] (Woody et al., 2019) which could be also interpreted as increased non-force-producing attachments due to rapid, fully reversible P_i_ release occurring before the power stroke (Robert-Paganin et al., 2020; Debold, 2021). In such a model, P_i_ rebinding decreases the probability of power strokes from the attached pre-force AM.ADP state rather than the lifetime of pre-power-stroke attachments or the size of the power stroke. Whether P_i_ is released before or after the power stroke, the observation of rapid power stroke upon strong attachment of myosin to actin implies that they are closely coupled fast equilibria, and the question is restricted to how the preceding strong binding of myosin to actin limits the first event. The sequential models analyzed in this study for *CS* did not involve the kinetics of myosin binding to actin. Time-resolved X-ray diffraction studies of contracting muscles have identified that the attachment of myosin heads to actin in the same type of conformation as that found during steady-state isometric contraction is the rate-limiting structural change for force development upon electrically stimulating intact muscle fibers (Reconditi et al., 2011). Both the pre-P_i_ release power stroke and the pre-power stroke P_i_ release scenario can result in high *CS*, provided that the reversible power stroke in the pre-P_i_ or the reversible P_i_ release in the pre-power stroke P_i_ release is strongly dependent on the preceding, rate-limiting, strong binding of myosin to actin.

In summary, the findings in this study demonstrate that the [P_i_]-modulated *k*_TR_-force relation is a sensitive probe of the coupling between the P_i_ binding step, reversal of the force-generating step, and transition limiting backward flux of cross-bridges from force-generating to non-force-generating states expressed by the rate constant *f* ^*–*^. The high *CS* observed in cardiac myofibrils, in combination with the simulations of *k*_TR_-force relations using various sequential models, indicates that P_i_ binding induced force reversal is strongly coupled to *f* ^–^. Additionally, previous studies on myofibrils have shown that a rapid decrease in [P_i_] to a low final [P_i_] induces a slow increase in force with a rate constant *k*_–Pi_ similar to *k*_TR_ indicating that P_i_ release coupled with force generation in the forward direction is strongly coupled to the rate-limiting transition *f* (Tesi et al., 2000; Stehle, 2017; Stehle and Tesi, 2017). Taken together, these findings indicate that the reversible force generation associated with P_i_ release cannot be decoupled from either the rate-limiting forward transition *f* or from the rate-limiting backward transition *f* ^*–*^. Consequently, this substantially limits models by excluding the potential contributions of other rate-limiting processes uncoupled from either reversible P_i_ release or force generation. Further research is needed to better define these rate-limiting transitions in the context of structural and chemical aspects of the myosin motor ATPase cycles.

## Data Availability

The raw data supporting the conclusions of this article will be made available by the authors, without undue reservation.
